# Q-RAI data-independent acquisition for lipidomic quantitative profiling

**DOI:** 10.1038/s41598-023-46312-8

**Published:** 2023-11-07

**Authors:** Jing Kai Chang, Guoshou Teo, Yael Pewzner-Jung, Daniel J. Cuthbertson, Anthony H. Futerman, Markus R. Wenk, Hyungwon Choi, Federico Torta

**Affiliations:** 1 Precision Medicine Translational Research Programme and Department of Biochemistry, Yong Loo Lin School of Medicine, National University of Singapore, Singapore, Singapore; 2 SLING, Singapore Lipidomics Incubator, Life Sciences Institute, National University of Singapore, Singapore, Singapore; 3 Department of Medicine, Yong Loo Lin School of Medicine, National University of Singapore, Singapore, Singapore; 4 Department of Biomolecular Sciences, Weizmann Institute of Science, Rehovot, Israel; 5 Agilent Technologies Inc., Santa Clara, CA USA

**Keywords:** Lipidomics, Mass spectrometry

## Abstract

Untargeted lipidomics has been increasingly adopted for hypothesis generation in a biological context or discovery of disease biomarkers. Most of the current liquid chromatography mass spectrometry (LC–MS) based untargeted methodologies utilize a data dependent acquisition (DDA) approach in pooled samples for identification and MS-only acquisition for semi-quantification in individual samples. In this study, we present for the first time an untargeted lipidomic workflow that makes use of the newly implemented Quadrupole Resolved All-Ions (Q-RAI) acquisition function on the Agilent 6546 quadrupole time-of-flight (Q-TOF) mass spectrometer to acquire MS2 spectra in data independent acquisition (DIA) mode. This is followed by data processing and analysis on MetaboKit, a software enabling DDA-based spectral library construction and extraction of MS1 and MS2 peak areas, for reproducible identification and quantification of lipids in DIA analysis. This workflow was tested on lipid extracts from human plasma and showed quantification at MS1 and MS2 levels comparable to multiple reaction monitoring (MRM) targeted analysis of the same samples. Analysis of serum from Ceramide Synthase 2 (CerS2) null mice using the Q-RAI DIA workflow identified 88 lipid species significantly different between CerS2 null and wild type mice, including well-characterized changes previously associated with this phenotype. Our results show the Q-RAI DIA as a reliable option to perform simultaneous identification and reproducible relative quantification of lipids in exploratory biological studies.

## Introduction

Lipids are a group of organic compounds with high hydrophobicity that play important biological roles, including cellular signalling, energy metabolism and as a structural component of cell membranes^1,2^. Lipidomics entails comprehensive identification and robust quantitation of lipids using analytical chemistry methods such as liquid chromatography mass spectrometry (LC–MS) and it has matured as an independent field of study underscoring the importance of metabolism in disease progression^1,2^. Typically, untargeted lipidomics focuses on the identification and relative quantitation of lipids for discovery approaches, while targeted lipidomics serves to identify and quantify a group or panel of known lipids with high sensitivity^3^.

In untargeted lipidomics there are two common analytical approaches. The first is direct-infusion MS (shotgun lipidomics), often performed using a nanospray source and a high-resolution mass spectrometer. Due to its speed, this is suitable for high-throughput studies and it has the advantage of allowing a reliable quantification, due to the same matrix effect experienced by all ions^4,5^. A disadvantage may be lower sensitivity and higher complexity of fragmentation spectra when compared to LC–MS. LC–MS based methods can perform better in terms of species coverage and sensitivity due to the chromatographic separation. Lipids with similar *m/z* values can be distinguished based on different retention times. In all these experiments, tandem MS (MS/MS) is essential for reliable identification. There are two ways to acquire MS/MS data in untargeted lipidomics: data dependent acquisition (DDA) and data independent acquisition (DIA). In DDA a full MS scan is conducted to obtain abundance-based precursor information of lipids in the sample, followed by fragmentation of the precursors to obtain MS/MS spectra of product ions. However, DDA approaches are sometimes ineffective in fragmenting low abundance compounds due to the preference given to the most abundant ones^6,7^. In addition, peak intensities of product ions are not quantitatively accurate as fragmentation is often triggered before the apex of the peak and there are insufficient data points along the chromatograms^6,7^. The disadvantages of DDA are then low reproducibility and incomplete coverage.

Recent advances in MS technologies have led to the development of DIA for comprehensive MS/MS data generation and targeted quantification at both precursor and product ion levels, thereby addressing some of the limitations of DDA, including biased selection of abundant ions for fragmentation. In DIA, all precursors within a selected mass range are scanned and consistently fragmented regardless of abundance. The major limitation of this technique is the high complexity of the spectra generated during fragmentation of co-eluting species, which makes lipid identification difficult. Ways of reducing this complexity include the use of well characterised LC separations and the use of consecutive quadrupole isolation windows^7,8^. A selective DIA methodology that was first reported by Gillet et al. utilizes sequential quadrupole isolation windows for acquisition of all theoretical mass spectra (SWATH) in proteomics applications, where the mass range of interest is divided into several subranges (or isolation windows) for fragmentation^9–13^. Generally, an overlap is included between windows, to ensure that all precursors in the desired mass range are selected for fragmentation^6^. When DIA is applied in direct infusion shotgun lipidomics, utilizing the MS/MS^ALL^ approach that is available in orbitrap mass spectrometers and latest hybrid quadrupole time-of-flight (Q-TOF) technologies, all precursors in the sample are essentially isolated across the entire mass range of interest in 1 m*/z* isolation windows, followed by fragmentation^10,14,15^.

Isolation window sizes can be customized depending on the application, with key considerations being the abundance of precursors, coelution of analytes and chromatographic peak widths. Since all precursors can be fragmented, larger window sizes would result in more precursors simultaneously entering the collision cell for fragmentation and hence resulting in increased complexity of MS2 spectra^8,16^. This causes issues particularly in high-throughput analyses, where numerous analytes co-elute and chromatographic peaks are narrow, with ion suppression and convoluted MS/MS with additional interferences that results in insufficient data points for accurate peak area calculation. The isolation window size and the number of windows is inversely related, where smaller window sizes would result in a larger number of windows to cover the entire mass range. These parameters also determine the total cycle time, which is derived from the MS1 and the MS2 acquisition rates and the number of windows. A general consensus is that there should be at least 10 data points (10 cycles) across the narrowest chromatographic peak for reliable quantification in chromatographic analyses^17^. For this reason, 25 m*/z* wide windows are commonly used in proteomics, and 10–20 m*/z* windows in lipidomics, to ensure a good balance between purity of MS/MS spectra and quality of quantification.

While the early generation of DIA methods was performed with fixed size windows, the need for reliable quantification has also spurred on the development of windows of variable sizes, optimized based on the precursor density across the entire mass range^18^. Smaller window sizes would be adopted for *m/z* regions with a higher density of precursors, while larger window sizes would be adopted for regions where precursors are less present, thereby reducing the loss of sensitivity^19^. This allows for the same total number of windows to be maintained, thereby ensuring that the cycle time is not compromised and enough data points can be captured for all ions of interest.

The choice between DDA and DIA methodologies also has implications on the downstream data analysis approach. For lipidomic data acquired in DDA mode, software with lipid identification capabilities such as MS-Dial, Lipid Annotator, LipidMatch, LipiDex, LipidXplorer, LipidBlast and others have been developed^20–25^. Most of these tools enable lipid identification by searching against a known database or spectral library using a combination of features such as retention time (RT), isotopic distribution, precursor mass and MS2 spectra. Others may use alternative approaches: MS-Dial utilises a decision tree algorithm within a rule-based annotation system, LipidXplorer implements user-defined fragmentation pathways described using the molecular fragmentation query language and Lipostar can work with a database-free rule-based fragmentation system^21,26^. Most software can read data from a variety of LC–MS instruments and methods. Recently, we have reported a new data processing software package, MetaboKit, to support seamless integration of DDA and DIA for identification and relative quantification, respectively with potential advantages over the alternatives, including the explicit MS/MS-based annotation of in-source fragments (ISFs) and the ability for the user to build and annotate an in-house spectral library based on product ion spectra and retention times^27^. The DIA module of MetaboKit performs a targeted extraction of MS2 fragments using spectral libraries obtained from DDA, which facilitates users to gradually build a customized database of MS2 spectra and decreases reliance on commercially or publicly available libraries for benchmarking in the long term.

Meanwhile, LC-based DIA methodologies for lipidomics have been most actively developed on SCIEX Triple-TOF instruments, with standardized workflows for identification and quantification^18^. Software packages such as MetDIA and MS-Dial have been developed to analyse the acquired MS2 complex data, where the fragmentation spectra are deconvoluted and benchmarked to spectral libraries in-built within the software^21,28^. Spectral deconvolution strategies are essential in DIA as the direct link between precursor and product ion spectra is lost and data become more complex due to higher background signals in the obtained spectra^29^. To optimize analytical methods, tools such as the SWATH Acquisition Variable Window Calculator by SCIEX, that calculate the best sequence of variable windows based on the density of precursors have been released^30^. However, these approaches have not been extensively validated for other instruments.

Herein, we report the development and application of Quadrupole-Resolved All Ions (Q-RAI) data from the Agilent 6546 Q-TOF, in combination with DDA-based library construction, as a novel alternative DIA analysis platform on Agilent LC–MS instruments. The proposed workflow is seamlessly supported by the MetaboKit software package. The Q-RAI acquisition mode is a SWATH-like data independent acquisition mode newly developed on the Agilent 6546 LC/Q-TOF, first reported for quantitative analysis of per- and polyfluoroalkyl substances (PFAS)^31^. However, this acquisition mode has not been tested for high throughput lipidomic applications and could provide an additional tool for simultaneous untargeted lipid identification and quantification at both MS1 and MS2 levels without significant loss in lipid coverage, particularly for users with this line of instruments. This DIA approach would be advantageous over conventional untargeted DDA workflows where MS2-based quantification is not applicable, especially for compounds in which the product ions offer quality ion chromatograms as good as or better than that of the precursor ion. In addition, it will also enable easier development of targeted assays from the product ion spectra of specific lipids of interest. To properly test this newly developed instrumental and data analysis workflow, we first analysed commercially available human plasma and then conducted a differential abundance analysis of murine serum samples from wild type and a Ceramide Synthase 2 (CerS2) null model.

## Materials and methods

### Reagents

1-Butanol was purchased from Merck (Darmstardt, Germany). Methanol, isopropanol and acetonitrile were purchased from Thermo Fisher Scientific (Waltham, Massachusetts, United States). Ammonium Formate (10M in water) was purchased from Sigma-Aldrich (St. Louis, Missouri, United States). All solvents were of HPLC grade. Pooled commercial human plasma obtained via EDTA whole blood from healthy subjects of European descent and mixed gender was purchased from Sera Laboratories International (West Sussex, United Kingdom). MilliQ water used for mobile phase preparation had a resistivity of 18 mΩ.

Most internal standards were purchased from AVANTI (Alabaster, Alabama, United States), including ceramide (Cer) d18:1/12:0, Cer m18:1/12:0, cholesterol (COH)-d7, glucosylceramide (HexCer) d18:1/12:0, dihexosylceramide (Hex2Cer) d18:1/12:0, lysophosphatidylcholine (LPC) 13:0, lysophosphatidylethanolamine (LPE) 14:0, phosphatidylcholine (PC) 13:0/13:0, phosphatidylethanolamine (PE) 17:0/17:0, phosphatidylglycerol (PG) 17:0/17:0, phosphatidylinositol (PI) 12:0/13:0, phosphatidylserine (PS) 17:0/17:0, plasmalogen phosphatidylcholine (PC-P) 18:0/18:1-d9, plasmalogen phosphatidylethanolamine (PE-P) 18:0/18:1-d9 and sphingomyelin (SM) d18:1/12:0. Other standards include acylcarnitine 16:0-d3, triacylglycerol (TG) 12:0/12:0/12:0 and TG 17:0/17:0/17:0 from Sigma-Aldrich, ganglioside GM3 d18:0/18:1-d3 and globotriaosylceramide (Hex3Cer) d18:0/18:1-d3 from Cayman (Ann Arbor, Michigan, United States), cholesteryl ester (CE) 18:0-d6 from CDN isotopes (Quebec, Canada) and diacylglycerol (DG) 15:0/15:0 from Santa Cruz Biotech (Dallas, Texas, United States).

### CerS2 serum samples

CerS2 null mice were generated as previously described, in accordance with the Animal Research: Reporting of In Vivo Experiments (ARRIVE) guidelines^32,33^. The experimental protocols were approved by the Weizmann Institute of Science’s Institutional Animal Care and Use Committee (IACUC), with animals treated according to the IACUC Animal Care Guidelines and the National Institutes of Health's Guidelines for Animal Care. Serum was isolated from wild type and CerS2 null mice at 26 weeks.

### Lipid extraction

Lipid extraction from commercial human plasma and mouse serum samples was performed using a variation of the Butanol-Methanol extraction method^34^. Briefly, 180 µL of Butanol-Methanol (1:1, v/v) spiked with internal standards were added to 20 µL of sample. The resultant mixture was vortexed for 10 s and then sonicated in a water bath at 4 °C for 30 min. Centrifugation was subsequently performed at 14,000 rcf at 4 °C for 10 min. The supernatant was transferred into MS vials for LC–MS analysis. To develop the DIA method, the lipid extracts from human plasma samples were pooled, mixed and aliquoted into MS vials.

Serum samples from five wild-type mice and five CerS2 null mice were used for DIA analysis. The sequence of serum samples for extraction and subsequent LC–MS analysis were obtained via random sampling on Microsoft Office Excel 2016. Aliquots from each serum extract were pooled and split across the analytical run for equilibration of the system (5 samples) and as technical quality control (QC) samples (6 samples analysed at regular intervals).

### LC–MS measurements

Lipid extracts were analysed on an Agilent 1290 Infinity II LC System coupled to the Agilent 6546 LC/Q-TOF system using a ZORBAX Eclipse Plus, C18, 95 Å, 1,8 µm, 2,1 × 50 mm (Agilent). Samples for MRM targeted analysis were measured with the same chromatographic system connected to the Agilent 6495 LC/TQ. The Agilent Data Acquisition Software Version 10.1 was used for all LC–MS analyses.

For the chromatographic runs, Water/Acetonitrile (6:4, v/v) + 10 mM Ammonium Formate was used as Mobile Phase A and Isopropanol/Acetonitrile (9:1, v/v) + 10 mM Ammonium Formate was used as Mobile Phase B, with an elution gradient starting at 0 min with 80% A and including: 2 min 40% A, 12 min 0% A, 14 min 80% A. The stop time was set at 15.8 min with a flow rate of 0.4 mL/min and a column temperature of 40 °C. An injection volume of 4 µL was used in positive mode and 8 µL in negative mode. For the dilution series, injection volumes of 0.25, 0.5, 1, 2, 4 and 8 µL were used.

The ion source parameters for DDA and DIA were as follows: Gas temperature 325 °C, Drying gas 10L/min, Nebulizer 20 psi, Sheath gas temperature 350 °C, Sheath gas flow 12L/min, Capillary voltage 3500 V, Nozzle voltage 1000 V, Fragmentor voltage 180 V, Skimmer voltage 45 V and Octopole RF 750 V.

#### DDA

For DDA, acquisition was set to auto MS/MS mode with a mass range of 300–1000 m*/z* for MS1 and 50–1000 for MS2 at acquisition rates of 4 spectra/s (Cycle Time 1.6 s). The iterative MS/MS function was used to increase the number of fragmented species and was set at 20 ppm tolerance with RT exclusion tolerance of 0.2 min. A narrow (~ 1.3 m*/z*) isolation width, collision energy of 20 V, 5 maximum precursors per cycle, precursor abundance-based scan speed of 10,000 counts/spectrum, 200 counts at 0.01% as the threshold for MS/MS, purity stringency of 100% and purity cut off of 30% were also used. Reference masses at 121.0599 m*/z* and 922.0098 m*/z* were used in positive mode and 112.9856 m*/z* and 966.0007 m*/z* in negative mode. Active exclusion was enabled for one repeat for 0.08 min, corresponding to about half peak width. Abundance dependent accumulation was used with a value of 10,000 counts with MS/MS maximum accumulation time limit set to the method cycle time and precursors that cannot reach target abundance were not rejected.

#### DIA

Acquisition was set to data independent acquisition mode with a mass range of 300–1000 m*/z* for MS1 and 50–1000 for MS2 at acquisition rates of 20 spectra/s for MS1 and 40 spectra/s for MS2. A fixed collision energy of 20 V was used.

The variable windows were obtained by analysing the acquired DDA data on the DIAwindow_calc module in the in-house developed MetaboKit software. The module first applies a feature detection step on the MS1 map to get a list of MS1 features. For each of these features, the density (number of features nearby) was calculated. The summed density for all features was approximately equal among all windows, subject to minimum and maximum window size allowable by the mass spectrometer. Finally, each window was extended by 0.5 m*/z* on both ends to ensure a 1 m*/z* overlap. The analysis was done using a window width of 10–100, 20 windows and mass range of 300–1000 m*/z*, with all other parameters kept as default.

Fixed and variable windows values used during method development are detailed in Tables S1 and S2 and the variable windows used for the mouse study are reported in Table S4. Variable windows in positive mode as depicted in Table S2 were used for the dilution series.

#### MRM

The triple quadrupole scan type was set to dynamic MRM with a delta EMV of 200 V (+) and 0 V (−) for analysis in positive mode. Time filtering was enabled with a peak width of 0.07 min and the cycle time was 900 ms. Ion source parameters were as follows: Gas temperature 250 °C, Gas flow 14 L/min, Nebulizer 35 psi, Sheath gas temperature 250 °C, Sheath gas flow 11 L/min, Capillary voltage 3000 V (+) and 4000 V (−), Nozzle voltage 1000 V (+ , −), High pressure RF 150 V (+ , −), Low pressure RF 60 V (+ , −).

### DDA data analysis

#### Lipid annotator

Raw DDA data files were analysed for identification of all lipid classes using settings as recommended by Koelmel et al.^20^: Q-Score ≥ 30, Mass deviation ≤ 10 ppm, Fragment Score ≥ 30, Total Score ≥ 60, Report dominant constituent if relative abundance differential ≥ 10%. All other parameters were kept as default.

#### MetaboKit

Agilent raw data files were first converted using MSConverter to mzML format. MSConverter is an open-source tool for conversion of raw data files to mzML format for data processing and is available at https://proteowizard.sourceforge.io/download.html^35^. DDA data were analysed on MetaboKit (2023/07/19 release) using the following settings: ms1to1 = 0.005 (amu), ms2to1 = 0.01 (Da), MS2_score = 0.5, min_peaks = 1 and RT_shift = 10. All other settings were kept as default, with MS/MS fragment matching fixed at 0.01 Da.

#### MS-dial

Agilent raw data files were first converted to abf format using AnalysisBaseFileConverter. DDA data were analysed on MS-Dial Version 4.72, with MS1 Tolerance 0.01 Da, MS2 Tolerance 0.025 Da, MS1 mass range 300–1000 m*/z* and MS2 mass range 50–1000 m*/z*. All other parameters were kept as default.

#### Lipostar

Raw DDA data files were directly analysed on Lipostar 2.1.2. Peak detection was done using a signal filtering threshold of 200 and m/z tolerance of 0.02 amu while all other parameters were kept as default. Identification was done using MS tolerance of 10 ppm and MS/MS tolerance of 20 ppm with all other parameters kept as default.

For the manual curation and filtering of software outputs, only [M + H]^+^ and [M + NH4]^+^ adducts were considered in positive mode and [M–H]^−^ and [M + HCOO]^−^ adducts in negative mode. Manual curation of all identified lipid species was done based on RT and manual inspection of the quality of spectral matching. Duplicate IDs for the same lipid species were also removed from the final set of readouts. For ambiguous lipid classes or species identified by the software but not previously observed in-house, quality of the spectral matching with the library was used as the main criterion for evaluation. We exercised a more conservative approach whereby at least two matching MS/MS peaks are required, followed by verification through in-house targeted tandem high-resolution MS/MS of these species using the same extracts. Lipids identified by the software but not present in the sample according to this manual inspection process were deemed to have failed filtering. For Lipostar outputs, identified lipids with an isotopic pattern score of 0 were also considered to have failed the filtering process.

### DIA data analysis

#### MetaboKit

Agilent raw data files were first converted to mzML format using MSConverter. DIA data were analysed with MetaboKit using the following settings: ms1to1 = 0.005, ms2to1 = 0.01, min_peaks = 1, RT_shift = 20. All other settings were kept as default, with MS/MS fragment matching of the software fixed at 0.01 Da.

#### Lipostar

Raw DIA data files were directly analysed on Lipostar 2.1.2. Parameters for peak detection and identification were the same as those used for DDA. Identified lipids with an isotopic pattern score of 0 were deemed to have failed the filtering process.

For both software, only [M + H]^+^ and [M + NH4]^+^ adducts were considered in positive mode and [M–H]^−^ and [M + HCOO]^−^ adducts in negative mode. Ambiguous species that were not identified in all triplicate injections, not previously observed and verified through in-house DDA or have doubtful RT were deemed to have failed the filtering process.

### MRM data analysis

Dilution series data were analysed with MassHunter Quantitative Analysis, Version 10.1 using a quantitative method specific for MRM data. Agile2 was used as the default integrator while for transitions with close multiple peaks, either General or Spectral Summation integrators were used. Manual inspection was also performed for all data following peak integration.

### Reference spectral libraries

For data analysis performed with MetaboKit, the HMDB, NIST, LipidBlast, Lipid Atlas and all publicly available libraries in the MS-Dial repository were used, together with an RT list and spectral library obtained from prior in-house DDA analysis. These libraries contained a total of 892,093 publicly available spectra in positive mode, 845,599 publicly available spectra in negative mode and 729 in-house spectra. However, all possible constituents for the same lipid sum composition are listed in the Lipid Atlas library in ascending numerical order and MetaboKit gives priority to the molecular composition with the highest match score, followed by the last entry listed in the spectral library when generating the outputs (e.g. SM 32:1 can be identified in negative mode based on the matching of the 659.5148 ion from formate loss and the 168.0416 phosphatidylcholine head group fragments but it is shown as SM 30:1;2O/2:0 in the output, which is the last entry for this particular sum composition in the Lipid Atlas library). Hence, all readouts were expressed as sum composition in the data for ease of interpretation. Readouts identified against the Lipid Atlas library had the sum composition computed using an in-house R script while readouts identified against the other libraries had the sum composition computed manually. Automated generation of sum composition lipid names by MetaboKit would be implemented in the foreseeable future but in the meantime, it is advisable for users to report the results at both levels to facilitate decision making with regard to the level of structural detail.

For MS-Dial analysis, all publicly available libraries in the MS-Dial repository were used for identification. In-silico libraries used in Lipid Annotator were taken from the MS-Dial repository using specific algorithms for transfer and assessment of quality control^20^.

### Statistical analysis

All calculations of R^2^, plotting of heatmaps and analysis for the mice study were done in RStudio (https://www.rstudio.com). Dot plots and visualization of lipid class distributions were generated using Graphpad Prism Version 8.4.3. Data for all other figures and tables were processed and analysed using Microsoft Office Excel 2016.

### Software availability

MetaboKit is an open-source command line tool publicly available at https://github.com/MetaboKit/MetaboKit. MS-Dial is also an open-source software^21^.

## Results and discussion

### Generation of spectral libraries

Conventional DIA workflows begin with MS/MS spectral library construction via DDA analysis. After generating an in-house MS/MS spectral library of lipids based on DDA acquisition of a commercial human plasma sample, we first tested the performance of different software packages for identification and quantification of lipids in our sample. Prior to data acquisition, we tested the impact of different collision energies (10 V, 20 V and 30 V) and observed that 20 V provided the best lipid coverage in both DDA and DIA (data not shown). Considering that the Q-RAI acquisition method is only configured with a single collision energy at the time of this publication, 20 V was selected for all our LC–MS analyses to maximize the lipid species coverage.

At this stage, the term quantification refers to peak area integration without normalisation by lipid standards, with the exception of the validation case study. In our experiment, ten iterative MS/MS injections of a pooled lipid extract were analysed using MetaboKit, Lipid Annotator, MS-Dial and Lipostar software in parallel, and the numbers of lipid species identified by individual tools were compared. MS-Dial was used for comparison as it is a commonly used software for processing of untargeted lipidomics data acquired using the DDA and DIA approaches and contains the spectral libraries used by MetaboKit. Lipid Annotator was used for this comparison as it was developed by Agilent specifically for the analysis of iterative DDA data files from Agilent Q-TOF instruments. Iterative MS/MS allows precursors selected for fragmentation in prior injections to be excluded from fragmentation in subsequent analysis of the same sample, allowing less abundant precursors to be detected and hence improving the overall coverage in DDA. On Agilent Q-TOF instruments, iterative selection of precursors can be enabled in the acquisition method whereas open-source iterative tools may be required for other platforms, such as AcquireX that is used for Thermofisher Orbitrap instruments^36^.

**Figure 1:**
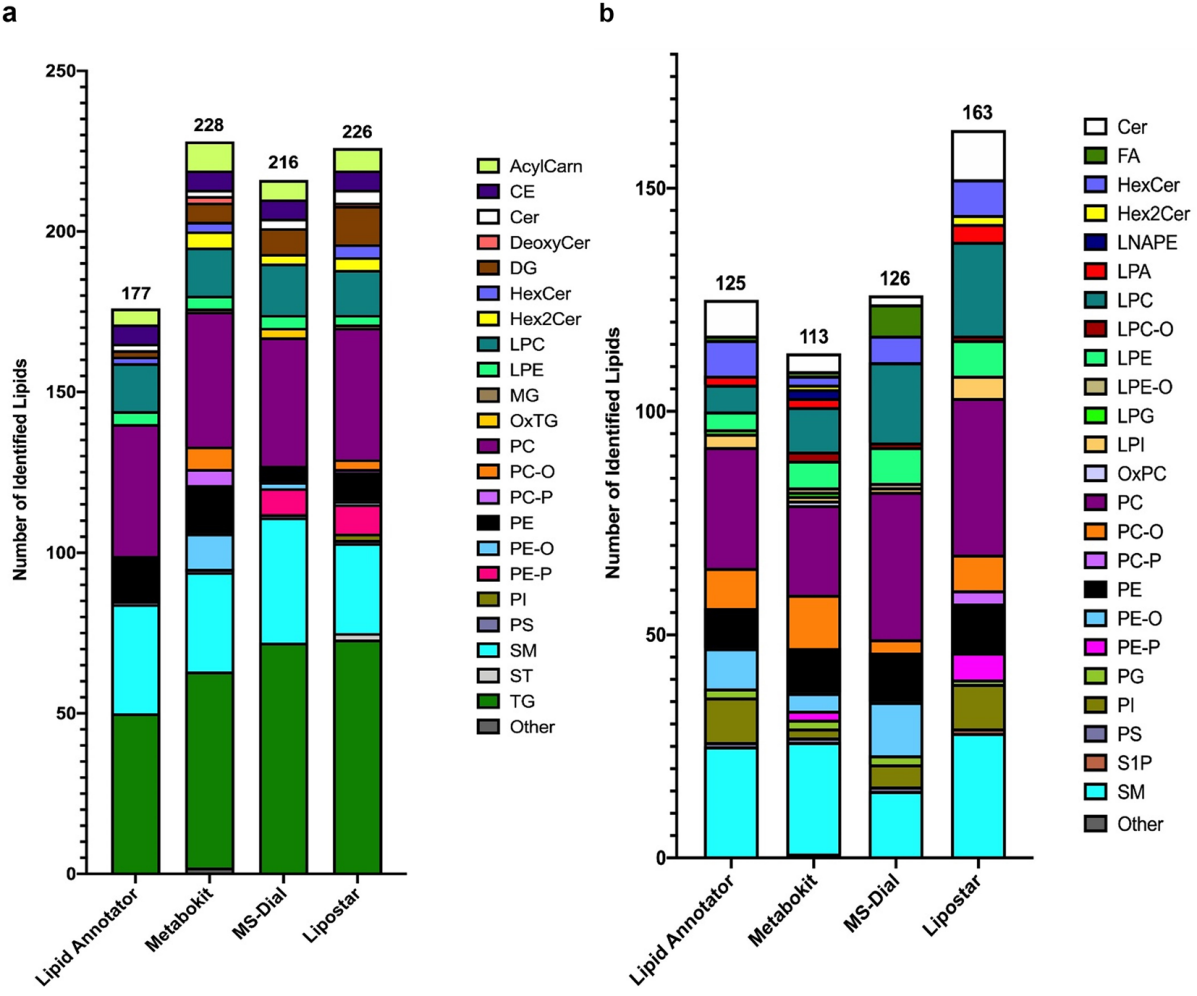
Identification of lipid species using Lipid Annotator vs MetaboKit vs MS-Dial vs Lipostar for (**a**) Positive Mode and (**b**) Negative Mode when analysing samples with iterative MS/MS (10 iterative injections) and DDA. In positive mode, MetaboKit gave the highest coverage with 228 lipids. In negative mode, Lipid Annotator, MetaboKit and MS-Dial gave comparable coverage while Lipostar gave the most identifications.

After manual curation, a total of 228 lipid species were identified with MetaboKit in the positive mode data, compared to 216 with MS-Dial, 177 with Lipid Annotator and 226 with Lipostar (Fig. 1a). Different tools showed variable coverage of molecular species identified in each class; more SM species were reported by MS-Dial, more DG species by Lipostar and more Hex2Cer, PC-Ps, PEs and ether lipids by MetaboKit. Across all software, TGs were the most commonly identified species, followed by PCs, SMs and LPCs.

In the negative mode data, Lipid Annotator, MetaboKit, MS-Dial and Lipostar gave a total of 125, 113, 126 and 163 species respectively (Fig. 1b). Similar to the positive mode analysis, some differences were observed in lipid class composition. MetaboKit was the only software that identified N-acyl-lysophosphatidylethanolamine (LNAPE) and lysophosphatidylglycerol (LPG) species, while Lipostar identified more ceramides, PCs, PEs and SMs. For Lipid Annotator, MetaboKit and MS-Dial in both polarities, a higher lipid coverage as compared to the traditional DDA approach was indeed observed with the iterative DDA approach and the use of five iterative injections was enough to reach the maximum number of species identified. (Fig. S1).

Although Lipostar gave the best identification results, it also reported 21,186 outputs that were manually filtered out in positive mode, which was significantly higher than other software ( Supplementary File 1). MS-Dial performed well but its report also contained a higher number of identifications that were filtered out in positive mode (334), compared to 73 and 203 in Lipid Annotator and MetaboKit, respectively (Supplementary File 1). Similarly, 15,762 identifications from Lipostar were filtered out in negative mode, compared to 86, 65 and 69 with MS-Dial, Lipid Annotator and MetaboKit respectively.

These differences in identification results could be due to the different identification algorithms, score thresholds, error rates and spectral libraries used by the different software. For instance, the Bayesian theorem algorithm combined with non-negative least squares for spectral deconvolution that is unique to Lipid Annotator could have resulted in a more conservative annotation, leading to lesser identified species in positive mode^20^. Conversely, the rule-based annotation system adopted by MS-Dial leads to a number of identifications generated without MS/MS evidence, which may have resulted in more false positives and reduced annotation confidence^21^. This would also imply that certain lipid classes are detected better by different tools. In turn, a balance needs to be achieved between identification numbers and stringency and any compounds of interest identified through this DDA approach would need to be further verified through tandem MS/MS and targeted analysis of the product ions.

To further assess the identification performance in DDA for MetaboKit, an analysis was also conducted using the same search space (spectral libraries) as MS-Dial, which yielded comparable performances (Fig. S2). These results showed that MetaboKit delivers comparably reliable identification performances with a coverage equivalent to other widely used tools, with the additional benefit of generating a customized MS/MS spectral library that includes product ion peak intensities and retention times. Overall, these findings suggest that MetaboKit is suitable for untargeted lipidomic analysis when using DDA and facilitates the generation and customization of data resources also for DIA analysis.

### DIA using the Q-RAI acquisition function for lipidomics

While initially developed as a quantitative tool for small molecules, we used the Agilent Q-RAI acquisition function as both a discovery and quantitation workflow for DIA analysis of lipids. Using the spectral library generated in DDA mode, a DIA approach was performed in both polarities on triplicates of the same pooled human plasma lipid extracts, using both fixed (20(+ 1) Da) and variable quadrupole windows. In this workflow, DDA was used as the primary mode of lipid identification while quantification in DIA was done only if tandem MS supported the lipid identification in DDA mode.

**Figure 2:**
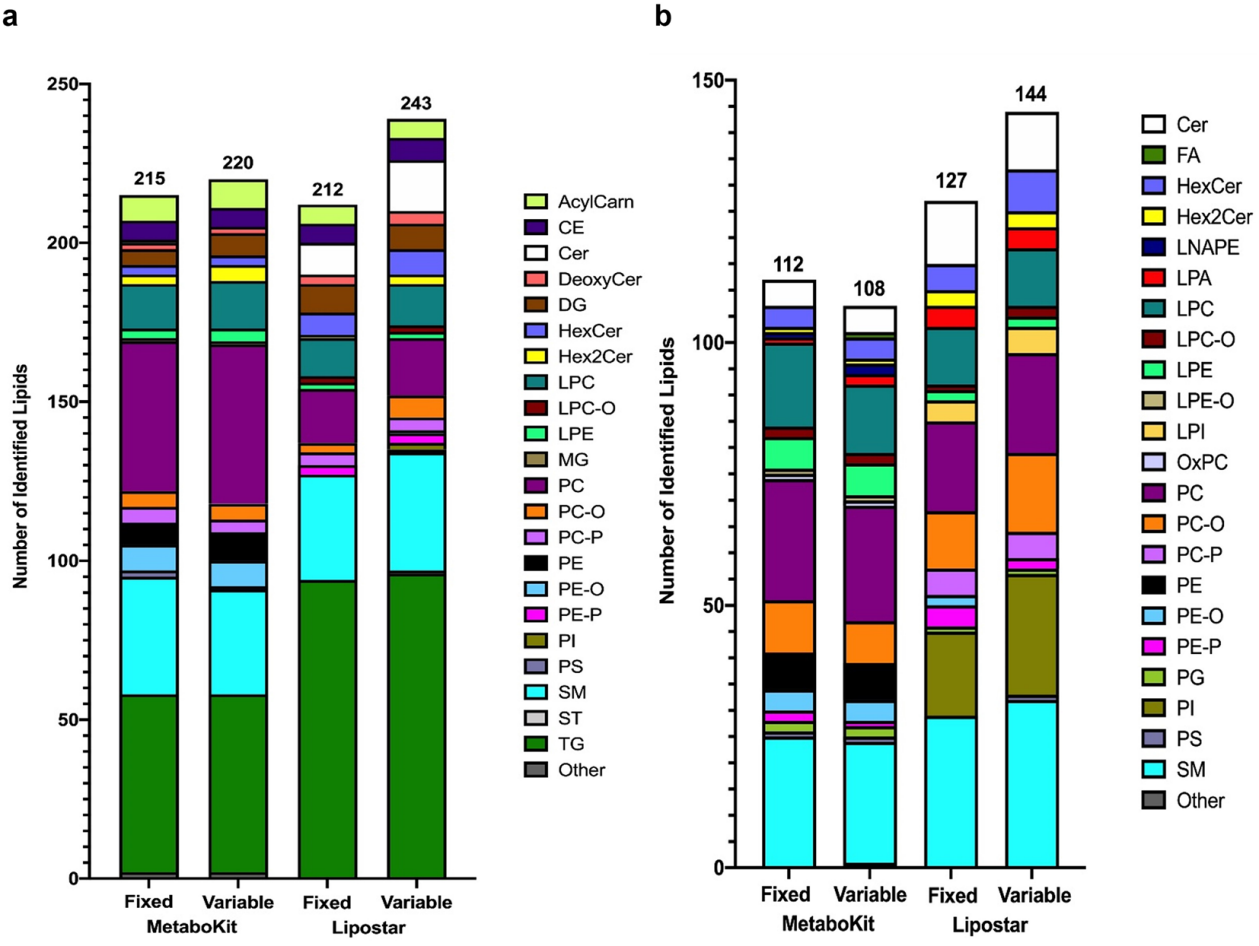
Number of lipid species identified when using DIA with the MetaboKit and Lipostar software for (**a**) Positive Mode and (**b**) Negative Mode using either fixed (20 (+ 1) Da) or variable windows. Lipostar gave marginally improved identification results, with the variable windows setup providing a slightly higher lipid coverage for both software in both polarities with the exception of MetaboKit in negative mode.

Among the lipids quantified in DIA positive mode, MetaboKit was able to extract quantitative data for 215 and 220 lipid species using fixed and variable windows, respectively (Fig. 2a, Supplementary File 2). Though these numbers show a slight reduction when compared to the 228 species identified with DDA positive mode using iterative MS/MS, these observations are expected since the quantification module requires that the ion chromatograms for the fragment ions in the DDA spectral library be retrievable for peak identification in DIA. Both types of isolation windows gave similar lipid class distributions, with TGs being the most represented, followed by PCs, SMs and LPCs. This trend is similar to the class distribution observed in DDA.

Among the lipids quantified in DIA negative mode, MetaboKit was able to extract quantitative data for 112 and 108 lipids using fixed and variable windows respectively (Fig. 2b, Supplementary File 2). Similar to the observations in positive mode, these numbers show a slight reduction when compared to the 113 species identified with DDA negative mode using iterative MS/MS. Detailed curation resulted in 145 and 124 MS1 features being filtered out in positive mode in fixed and variable windows respectively and 42 and 45 MS1 features being filtered out in negative mode in fixed and variable windows respectively. These results indicate that peak areas were associated to most of the lipid entries in the spectral library built with the previous DDA analysis and the targeted DIA extraction of MS2 fragments by MetaboKit enables MS2 quantitation of most lipid species identified in DDA.

When analysing the same data files with Lipostar, which also supports lipid identification using DIA data, 212 and 243 lipid species were identified in positive mode using fixed and variable windows respectively, while 127 and 144 lipid species were identified in negative mode (Fig. 2). Manual curation resulted in 12,662 and 14,313 MS1 features being excluded in positive mode in fixed and variable windows respectively and 4,505 and 5,327 MS1 features being excluded in negative mode. As this type of data generation is not yet widely adopted on Agilent instruments, the input file format was not compatible with MS-Dial (at least when this manuscript was generated) and we were not able to optimally process Q-RAI Agilent data files using this bioinformatic tool. In addition, Lipostar does not provide MS2 quantification in the software outputs and hence we were unable to compare MS2 quantification performances.

In terms of quantification, when using MS1 data obtained with DIA in both polarities, 98–99% of identified features showed RSD < 30%. Even though the fixed and variable windows approaches gave similar lipid coverages, the variable windows approach provided more reproducible results at the MS2 level, as seen from a higher number of fragments with RSD < 30%. The variable windows setup also generated more quantifiable MS2 fragments, as expected and previously reported^6,37^. More than 90% of MS1 signals had at least one corresponding MS2 fragment with RSD < 30%, confirming that a reliable quantification of lipids using their product ions is one of the advantages of the DIA approach. As expected, the RSD values at both MS1 and MS2 levels decreased exponentially with increasing abundance, with MS2 quantitation giving higher RSD than MS1 (Fig. S3). Table 1 summarizes the quantification data.

**Table 1:** Descriptive statistics for the comparison between MS1 and MS2 measurements in DIA mode.

	Positive Mode		Negative Mode	
	Fixed Windows (20 (+ 1) Da)	Variable Windows	Fixed Windows (20 (+ 1) Da)	Variable Windows
MS1				
Total Number of Features	215	220	112	108
Number of Features with RSD < 30%	209 (97.21%)	217 (98.64%)	112 (100%)	107 (99.07%)
Median RSD for Features with RSD < 30%	2.43	2.37	3.04	2.06
Number of Features with RSD > 30%	6 (2.79%)	3 (1.36%)	0 (0%)	1 (0.93%)
MS2				
Total Number of Fragments	1982	2038	1078	1040
Number of Fragments with RSD < 30%	1150 (58.02%)	1317 (64.62%)	471 (43.69%)	523 (50.29%)
Median RSD for Fragments with RSD < 30%	8.44	7.16	10.96	9.45
Number of MS1 Features with MS2 Fragments of RSD < 30%	210 (95.45%)	217 (98.64%)	109 (97.32%)	107 (99.07%)
Number of Fragments with RSD > 30%	573 (28.91%)	540 (26.50%)	316 (29.31%)	301 (28.94%)
Number of Non-Quantifiable Fragments	259 (13.07%)	181 (8.88%)	291 (26.99%)	216 (20.77%)

These results show that the Q-RAI DIA approach coupled with MetaboKit processing is a feasible approach for semi-targeted quantification of lipids in both MS1 and MS2. For this purpose, a variable windows approach may be more suitable, due to its higher reproducibility and sensitivity (Table 1). The improved identification performances using variable windows, as seen from the Lipostar readouts, also showcases an added benefit of this methodological approach but would require further testing of identification algorithms in MetaboKit and other tools.

### Comparison between Q-RAI DIA and MRM modes for quantification of lipids

To better evaluate the quantification performance of the Q-RAI DIA methodology, a dilution series using pooled plasma extracts was analysed using variable windows in DIA positive mode with the Q-TOF and, at the same time, with a targeted MRM method using a QQQ. While our DIA approach was not expected to outperform MRM due to different sensitivity performances, the rationale was to use the MRM approach as a benchmark and compare different quantification strategies. Various lipid amounts on column were measured by injecting serially increasing volumes: 0.25, 0.5, 1, 2, 4 and 8 µL. Table 2 and Supplementary File 3 summarizes the obtained results.

**Table 2:** Descriptive statistics for the comparison between measurements acquired on a Q-TOF by MS1 (DIA) and MS2 (DIA), or on a QQQ by MRM, for a six-point dilution series of a lipid extract from commercially available human plasma.

	Injection Volume (μL)					
	0.25	0.5	1	2	4	8
DIA MS1						
Total Number of Features	106					
Median RSD (%)	3.80	2.35	1.82	1.09	1.08	0.92
Number of Features with RSD > 30%	1 (0.94%)	0 (0%)	0 (0%)	3 (2.83%)	2 (1.89%)	1 (0.94%)
Median R^2^	0.990					
Number of Features with R^2^ < 0.8	5 (4.72%)					
DIA MS2						
Total Number of Quantified Fragments	651 out of 1007 (64.65% of all fragments)					
Median RSD (%)	15.15	11.02	8.27	6.06	5.42	4.74
Number of Fragments with RSD > 30%	167 (25.65%)	108 (16.59%)	73 (11.21%)	61 (9.37%)	37 (5.68%)	58 (8.91%)
Median R^2^	0.957					
Number of Fragments with R^2^ < 0.8	143 (21.97%)					
MRM						
Total Number of Transitions	287					
Median RSD (%)	5.74	1.71	1.41	1.68	1.38	5.51
Number of Transitions with RSD > 30%	0 (0%)	0 (0%)	0 (0%)	0 (0%)	0 (0%)	0 (0%)
Median R^2^	0.997					
Number of Transitions with R^2^ < 0.8	0 (0%)					

**Figure 3:**
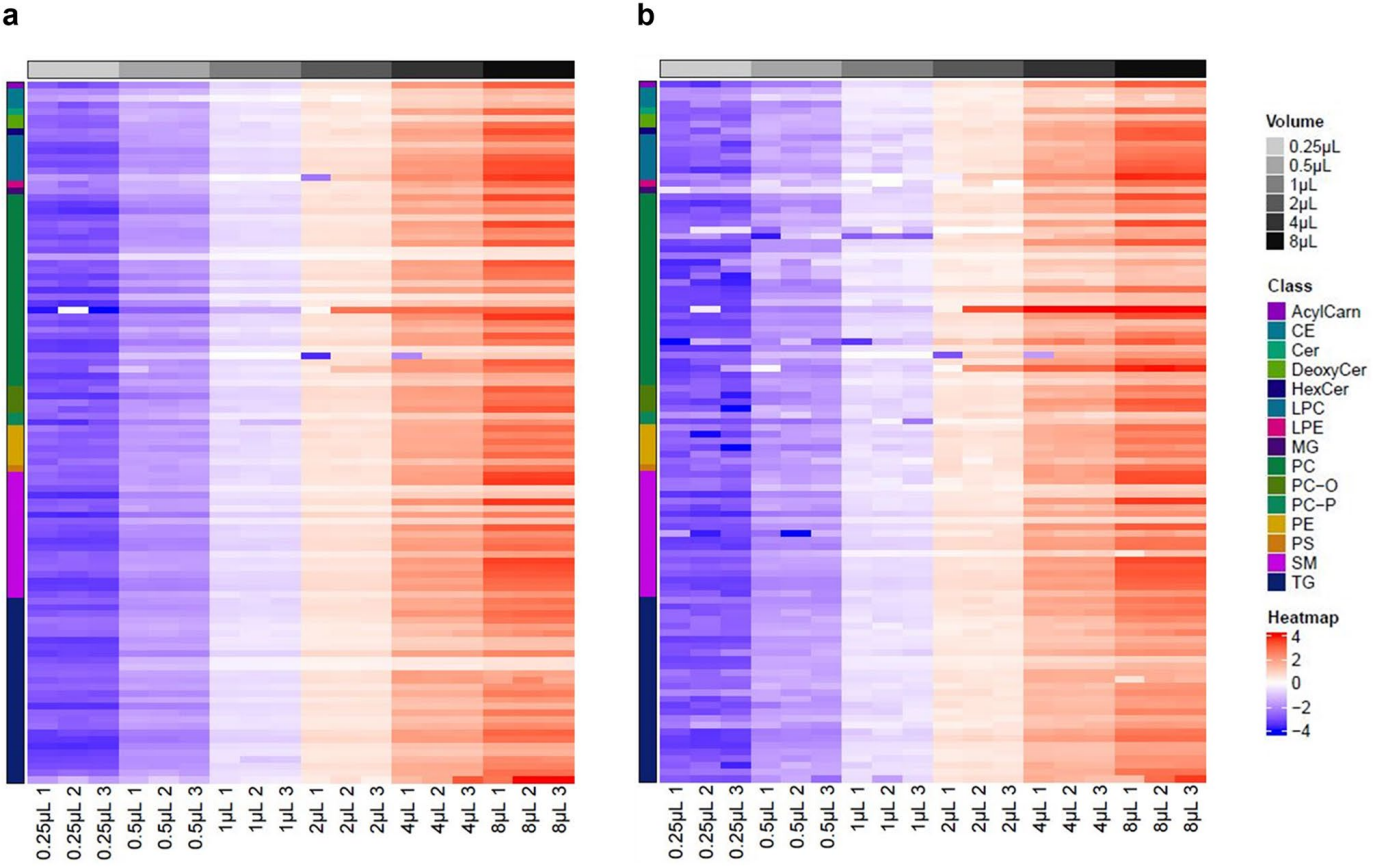
Heatmaps of normalized median-centred fold changes of DIA MS1 (**a**) and DIA MS2 (**b**) for 106 lipid species identified and quantified in dilution series of lipid extracts from commercial human plasma. A distinct increase in concentration, directly proportional to increasing injection volumes (grey to black), is shown for all lipids measured, for both DIA MS1 and DIA MS2 acquisitions.

When using DIA, similar results were obtained for the RSD at MS1 and MS2 level, with 8 µL injection volume giving the lowest RSD values followed by 4 µL (Table 2). However, saturation was evident at injection volumes above 4 µL. In view of this, the R^2^ for all outputs was calculated only up to an injection volume of 4 µL. A good linear response was observed, whereby MS1 showed R^2^ of 0.988 and for MS2 this was found to be 0.978 (Table 2). For the 106 lipid species monitored in the DIA dilution series data, heatmaps of the normalized median-centred fold changes showed proportional increases in the abundance for all lipids according to the injection volume, both at the MS1 (Fig. 3a) and MS2 (Fig. 3b) level.

To further investigate the potential use of MS2 for quantification, we calculated the Pearson’s correlation between the 651 generated DIA MS2 fragments and their corresponding MS1 precursors, obtaining a median Pearson's R^2^ of 0.973 with only 126 fragments (19.35%) having R^2^ < 0.8 (Table S3). As the best candidates for quantification for each of the 106 identified lipid species, the DIA MS2 fragments with the highest R^2^ for each MS1 feature were chosen. When considering these 106 MS2 fragments, the median R^2^ increased to 0.997 with no fragments having R^2^ < 0.8 (Table S3).

A similar comparison between MRM and DIA showed that across all injection volumes, the median RSD and the percentage of fragments with RSD > 30% were the lowest when using MRM and highest when using DIA MS2 (Table 2). As described for DIA, saturation was observed for injection volumes > 4 µL, while MRM gave the best linearity values, with no transitions having R^2^ < 0.8 and a median R^2^ of 0.997 (Table 2). In light of this, different injection volumes of 4 µL and 8 µL were deemed optimal for DIA MS2 quantification in positive and negative mode respectively, due to the different sensitivity for different polarities and based on the lowest median RSD calculated when injecting different volumes.

When using only the lipid species commonly found in the DIA and MRM methods, similar results were obtained in terms of RSD and R^2^ (Figs. S4, S5). 4 signals out of 88 showed a Pearson’s R^2^ < 0.8 when considering MRM vs DIA MS2, higher than the 2 signals out of 106 when comparing MRM and DIA MS1 (Fig. S6). When selecting the DIA MS2 fragment that showed the best correlation with DIA MS1, a higher correlation value with the corresponding MRM data was also observed, improving the results obtained when using the same MS2 fragment for both DIA and MRM (Fig. S7). Comparing the lipid species in common between DIA MS1, DIA MS2 and MRM also saw a reproducible linear increase of signal with injection volume for all three approaches (Fig. S8).

The differences observed in the reproducibility (RSD) and linearity suggest that quantification performances are better in MRM mode. Although we did not observe a clear superiority of the Q-RAI DIA workflow, our results show that DIA MS1 features, DIA MS2 fragments and MRM transitions are highly correlated. This result is somewhat expected, considering the high sensitivity and reproducibility of MRM methodologies. However, considering that prior knowledge of lipids of interest is required for MRM, together with the time and effort required to develop a suitable targeted panel, the Q-RAI DIA approach is a valid choice for simultaneous untargeted lipid identification without prior knowledge and reasonably precise MS2 quantification of identified lipid species.

Due to the presence of multiple MS2 fragments for each lipid, this DIA workflow can also be used to identify the best quantifier ions for targeted lipidomics optimization. User discretion might be advisable in terms of selecting the most representative MS2 fragments to quantify each MS1 feature for different applications. As shown in our results, Pearson’s correlation may be used as a criterion for selection of the best performing MS2 fragments for improvement of existing MRM lists. In addition to this, a variety of other criteria such as RSD, dot product score and linearity assessment may be used for the selection of either MS1 or MS2 ions. In general, MS1 measurements may have higher precision while MS2 measurements would be more sensitive and with their fragmentation information could serve as a more accurate quantifier, but this would be dependent on the analytes. For instance, ionization efficiency is dependent on numerous factors such as basicity of the analytes and solvents, relative intensities of the analytes, type of LC–MS instrument and experimental conditions, which could all contribute to deviation in MS1 quantification. The fragmentation yield is largely dependent on collision energy and may not be the optimal one for all analytes when using a constant value^38,39^. As such, one might expect to observe less background noise for MS2 and more variability in MS1 quantitation (although a larger cohort would have to be used to further evaluate this aspect).

### Case study: lipidomic analysis of blood serum from Ceramide Synthase 2 null mice

Ceramide Synthase 2 (CerS2) is one of the six Ceramide Synthase (CerS1-CerS6) enzymes, which are distinguished by their use of distinct sub-sets of acyl CoAs for N-acylation of the sphingoid base^40^. CerS2 uses mainly C22 and C24 acyl CoAs, thus generating very long acyl chain (VLC)-dihydroceramides and ceramides—the backbones of all sphingolipids (SLs)^32,33^. Lack of CerS2 results in a significant reduction of all VLC-SLs and a compensatory higher production of long chain C16 and C18 SLs^32,33^. The depletion of VLC-SLs changes membrane properties, disrupting membrane domains and their properties. CerS2 null mice show defects in insulin resistance (by inhibition of insulin receptor translocation), fatty acid uptake (by CD36 mislocalization and FATP5 down-regulation) and gap junction dysfunction^41^. Increased levels of C16-ceramides and sphinganine inhibit the mitochondrial respiratory complex IV, causing chronic oxidative stress^42^. Hence, perturbation of the expression of CerS2 results in a reduction of C22- and C24-containing SLs and could induce a possible significant change in the concentrations of other lipid classes, which has not been extensively studied, via a possible regulatory effect of the affected sphingolipid species. Application of our newly developed DIA workflow in this context would both confirm the effect of CerS2 suppression on the SL levels but also shed light on the influence of SL perturbations on other lipid pathways.

The DIA workflow was applied to the identification and quantification of lipids in serum samples from wild type (WT) and CerS2 null male mice. Five iterative injections of a pooled extract of all the murine samples were analysed in both polarities in DDA mode for generation of an in-house spectral library of lipids in mouse serum. Study samples, from five WT and five CerS2 null mice, were then injected in triplicates in DIA acquisition mode, using variable windows calculated by MetaboKit (Table S4).

Similar to what was found in human plasma, DIA MS1 provided a more reproducible quantification than MS2 on average, based on the metrics calculated for the pooled quality control (QC) samples. The median RSD associated with DIA MS1 measurements was 7.41 and 5.23 across all features in positive and negative mode respectively, while for DIA MS2 the median RSDs were 17.72 and 18.13 (Table 3). In total, 244 and 143 lipid species were quantified in DIA positive and negative mode in the QC samples (Table 3).

**Table 3:** Descriptive statistics of all transitions quantified in DIA mode for the QC samples (n = 6) of the CerS2 null mice study.

	Positive Mode		Negative Mode	
	DIA MS1	DIA MS2	DIA MS1	DIA MS2
Total Number of Quantified Features/Fragments	244	1844 out of 2505	143	755 out of 1461
Median RSD (%)	7.41	17.72	5.23	18.13
Number of Features/Fragments with RSD > 30%	4 (1.64%)	552 (29.93%)	1 (0.70%)	195 (25.83%)
Number of Features/Fragments with RSD 20–30%	7 (2.87%)	281 (15.24%)	2 (1.40%)	152 (20.13%)
Number of Features/Fragments with RSD < 20%	233 (95.49%)	1011 (54.83%)	140 (97.90%)	408 (54.04%)

**Figure 4:**
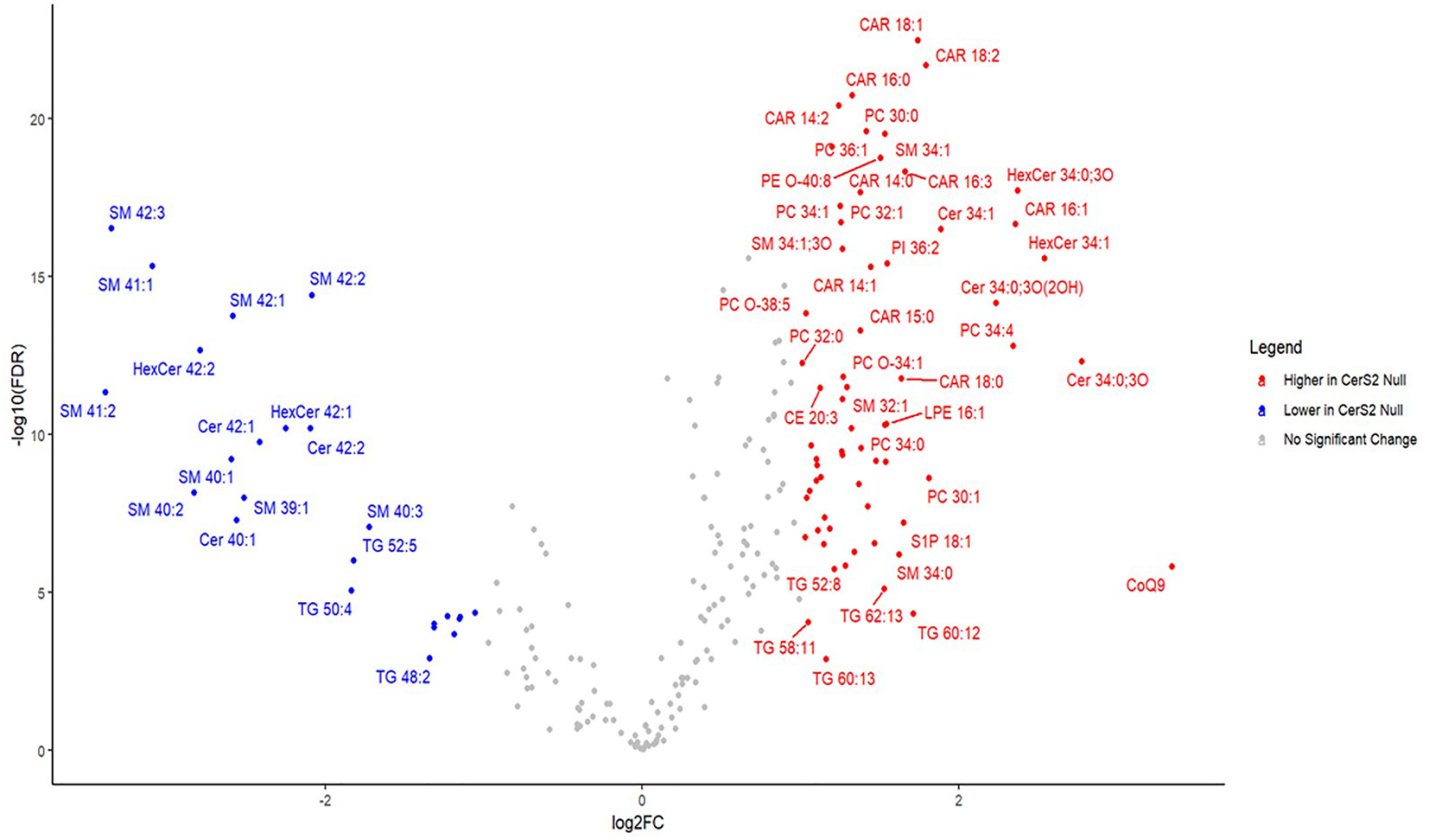
Volcano plot representing all lipids quantified in WT and CerS2 null mice serum using a Q-RAI DIA workflow. 88 lipids showed significantly different concentrations between WT and CerS2 null (FC > 2, FDR < 0.05). As expected, very long chain sphingolipids (C22-24) were present at significantly lower levels in CerS2 null mice, while acylcarnitines, phosphatidylcholines (PCs) and long chain sphingolipids (C16-18) were significantly higher in CerS2 null mice.

Lipid species with (1) MS1 and at least one MS2 fragment with RSD < 30% in the QC samples and (2) Pearson’s R^2^ > 0.8 between MS2 and MS1 in all samples were retained for analysis. Considering both polarities, a total of 173 lipid species passed the filtering criteria in positive mode and 119 in negative mode (Table S5). For these lipids, missing values in the mouse serum samples were then replaced using the outputs in the quant_fill_all text file and differential abundance analysis was conducted (Supplementary File 4). Choosing either the MS1 or the MS2 readout with the lowest RSD across both polarities as a representative result for each lipid, we found that 88 lipids had a significantly different abundance between WT and CerS2 null serum (Fig. 4). For some of the lipid species, after MS2-based quantification their levels were significantly different between WT and CerS2 null sera even when MS1 measurements although showing the same trend, did not reach statistical significance (Fig. S9a,c, Supplementary File 5). This indicates that in some cases DIA MS2 may be more sensitive for quantification. Nevertheless, for both polarities, most lipids quantified in the two groups were confirmed to be significantly different at both MS1 and MS2 levels (Fig. S9a,c). The heatmap of normalized median-centred lipid fold changes in the samples also shows distinct clustering based on lipidomic expression profile at both MS1 and MS2 levels (Fig. S10). These clusters revealed the presence in the serum of CerS2 null mice of lower levels of VLC-SLs and TGs but higher levels of other species, such as LPCs, acylcarnitines and ether-PC and -PE. This approach also further emphasized the effectiveness of using Pearson’s correlation in addition to RSD as a criterion for the selection of either MS1 or representative MS2 fragments from our Q-RAI workflow for semi-quantification.

**Figure 5:**
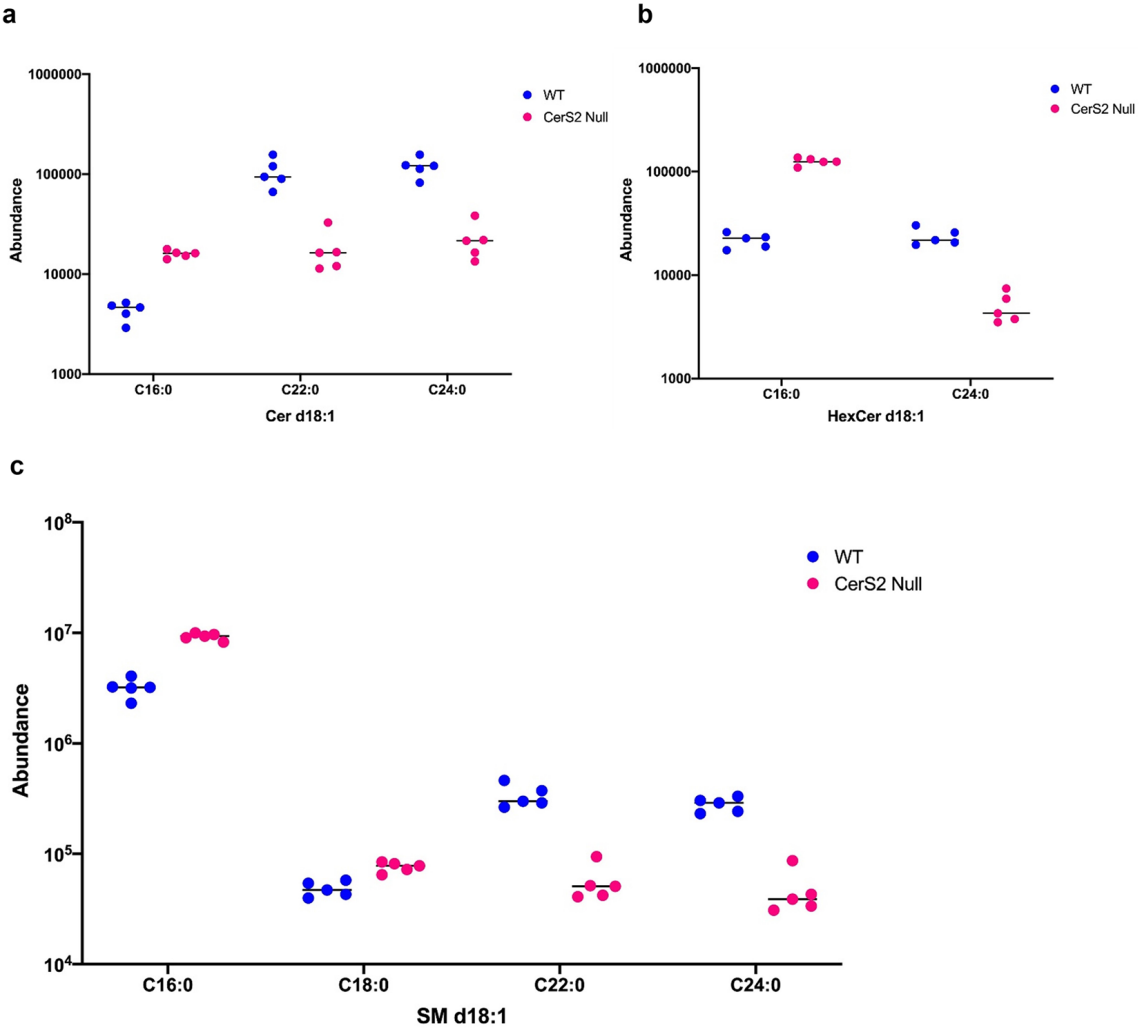
Dot plot of abundance values for long chain FA (C16 and C18)- and very long chain FA (C22 and C24)-containing sphingolipid species in WT (n = 5) and CerS2 null (n = 5) mice, obtained using the developed Q-RAI DIA workflow. Confirming what was previously reported, very long chain ceramides (**a**), hexosylceramides (**b**) and sphingomyelins (**c**) were significantly decreased in CerS2 null mice, while long chain species in the same lipid classes were significantly increased in CerS2 null mice (FC > 2, FDR < 0.05).

Reflecting the specificity of the CerS2 enzyme, the most significant changes were registered on SLs, such as ceramides and SMs carrying C22 and C24 fatty acyl chains, having significantly lower levels in CerS2 null mice (Fig. 5)^32,33^. At the same time, for the previously mentioned compensatory mechanism, the long chain C16- and C18-containing species were increased (Fig. 5)^32,33^. For most of these SL, significantly different concentrations were found both at MS1 level and for all fragments in MS2 (Supplementary File 5). These findings on SL are also consistent with the MRM data previously published by Pewzner-Jung et al.^32,33^ and confirmed the reliability of our DIA approach for quantification.

When considering other lipid species, an increase was observed for Coenzyme Q9 (CoQ9), several acylcarnitines and PCs, while TG species with saturated or monounsaturated fatty acyl chains decreased. Dysregulation of acylcarnitine levels suggests an impaired function of the mitochondria caused by incomplete β-oxidation of lipids, possibly leading to elevated oxidative stress, as previously shown in the same animal model^43,44^. As a compensatory mechanism, increased production of CoQ9 might occur to coordinate the assembly of the electron transport chain and promote antioxidative function within the mitochondria^45^. This informative finding was only allowed by the Q-RAI DIA untargeted approach, as this is not a molecule usually covered by lipid panels. An increased level of C16 sphingolipids can also induce similar mitochondrial defects, which could generate an increase in the TG levels as a buffering system for the increase in toxic free fatty acids^43,46,47^. Conversely, C22-C24 ceramides can cause dysregulation of hepatic CD36/FAT expression, thereby resulting in reduced hepatic accumulation of TGs and reduced secretion into plasma as a complex with very low-density lipoproteins (VLDLs) or chylomicrons^48–50^. Another interesting finding is the negative correlation existing between VLC-SL levels and ether lipids, which has been observed earlier by other investigators in cell models^51,52^. This effect seems to be due to SL and ether lipids having similar functions in the cell membrane. Although the reason for this co-regulation is not known, from our results we can hypothesise that this might be mainly governed by the SL species containing VLC FA, as in our system the long chain SL changed in the same direction as the ether species.

More in general, 38 and 44 lipids were found to have significantly different levels between WT and CerS2 null samples in positive mode and negative mode respectively (Fig. S9b,d). These findings indicate the success of the Q-RAI DIA approach in elucidating the possible pivotal role of CerS2 as a regulatory gene in liver homeostasis and general lipid metabolism, thereby illustrating the potential of this methodology for high-throughput lipidomic screening in biological applications.

## Conclusion

In summary, we developed for the first time a DIA workflow for lipidomics using Q-RAI acquisition on the Agilent 6546 LC/Q-TOF instrument. Following data acquisition, MetaboKit provides robust functionalities in untargeted lipid identification for DDA analysis and targeted quantification for DIA data, at both the MS1 and MS2 levels, using a customized spectral library. The availability of multiple MS2 fragments for each identified MS1 feature in the DIA readouts provides an option for the user to select representative MS2 fragments of interest for each compound, using pre-defined objective quality metrics, which will be particularly applicable as a digital footprint for development of in-house targeted methods or for small scale biological studies. This solution may potentially be extended to larger scale applications and analysis of polar metabolites in the near future.

### Supplementary Information

Supplementary Information 1.

Supplementary Table S1.

Supplementary Table S2.

Supplementary Table S3.

Supplementary Table S4.

Supplementary Table S5.

## Data Availability

Metabolomics data was deposited to the EMBL-EBI MetaboLights database (https://doi.org/10.1093/nar/gkz1019, PMID:31691833) with the identifier MTBLS8097. The complete dataset can also be obtained by contacting the corresponding author.
