# Author Correction: Q-RAI data-independent acquisition for lipidomic quantitative profiling

**DOI:** 10.1038/s41598-024-55396-9

**Published:** 2024-02-28

**Authors:** Jing Kai Chang, Guoshou Teo, Yael Pewzner-Jung, Daniel J. Cuthbertson, Anthony H. Futerman, Markus R. Wenk, Hyungwon Choi, Federico Torta

**Affiliations:** 1 Precision Medicine Translational Research Programme and Department of Biochemistry, Yong Loo Lin School of Medicine, National University of Singapore, Singapore, Singapore; 2 SLING, Singapore Lipidomics Incubator, Life Sciences Institute, National University of Singapore, Singapore, Singapore; 3 Department of Medicine, Yong Loo Lin School of Medicine, National University of Singapore, Singapore, Singapore; 4 Department of Biomolecular Sciences, Weizmann Institute of Science, Rehovot, Israel; 5 Agilent Technologies Inc., Santa Clara, CA USA

Correction to: *Scientifc Reports* 10.1038/s41598-023-46312-8, published online 07 November 2023

In the original version of this Article, the Acknowledgements section was incomplete. It now reads:

“This work was supported by the Israel Science Foundation-National Research Foundation of Singapore (NRF) collaborative grant (Grant Number 3172/19), the Singapore MOE grant (Grant Number T2EP20121-0016), the Singapore NMRC grant (Grant Number CG21APR1008) and NUS-Agilent Hub, and by the NRF2019-NRF-ISF003-3172 grant.”

The original Article has been corrected.

