# Supplementary material for: Q-RAI data-independent acquisition for lipidomic quantitative profiling
Source: Sci Rep. 2023 Nov 7;13:19281. doi: 10.1038/s41598-023-46312-8 (PMC10630469; doi:10.1038/s41598-023-46312-8)
Supplement: Supplementary file 1 — Supplementary Information 1. [file 41598_2023_46312_MOESM1_ESM.docx]

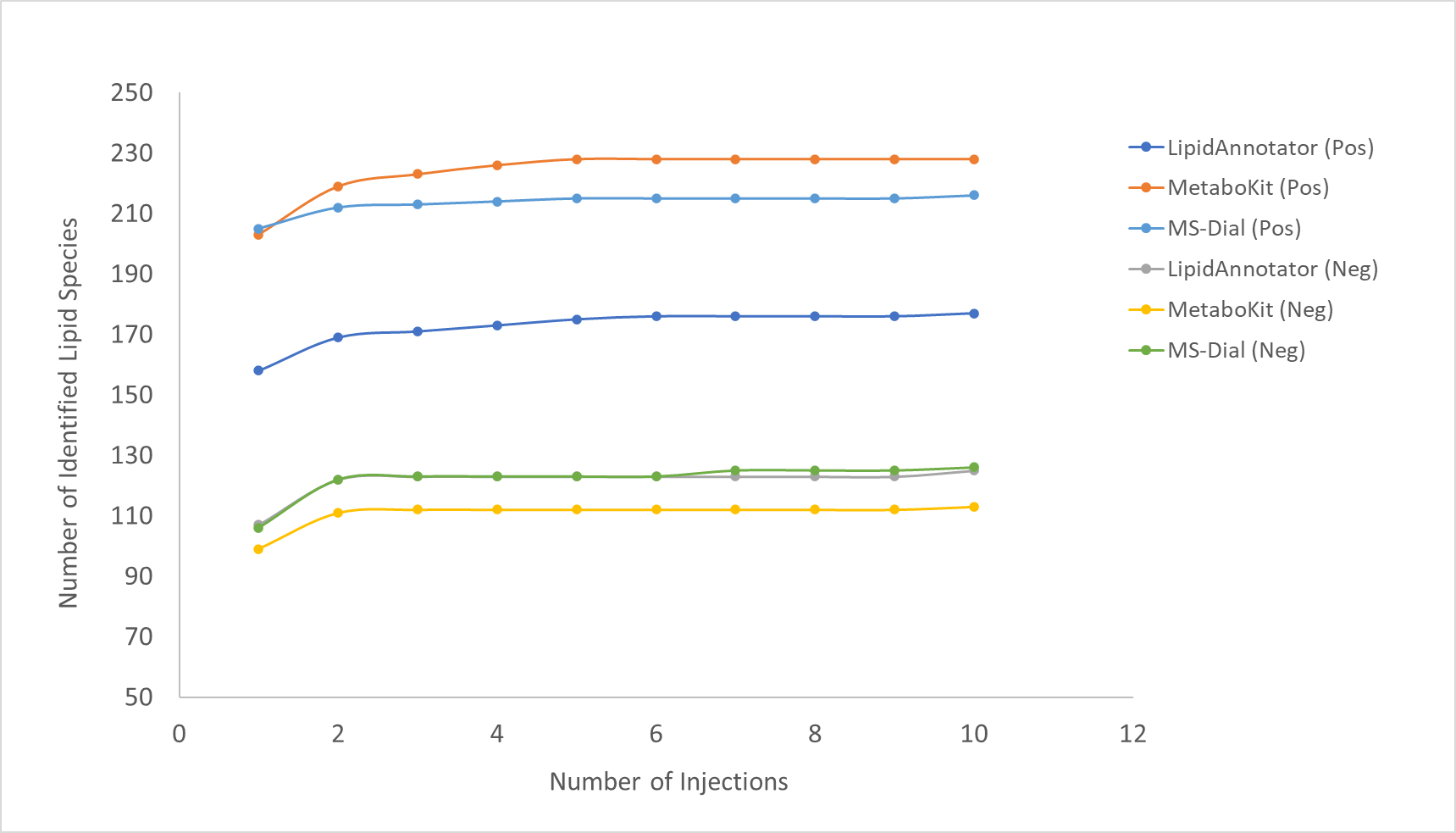


**Figure S1:** Increase in the number of annotations over injections when using iterative exclusion for DDA data analysed on Lipid Annotator, MetaboKit and MS-Dial in both positive and negative modes. In these conditions, lipid coverage does not improve beyond 5 injections.

**a b**

**
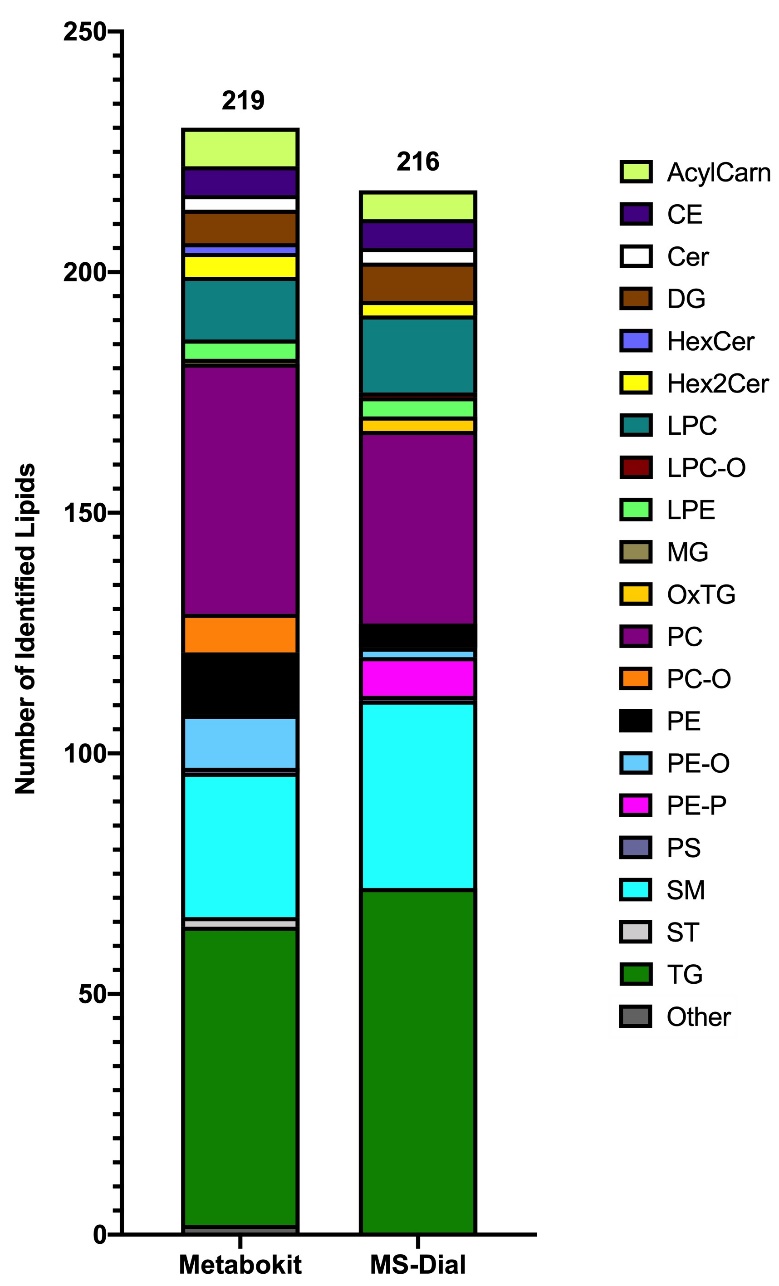

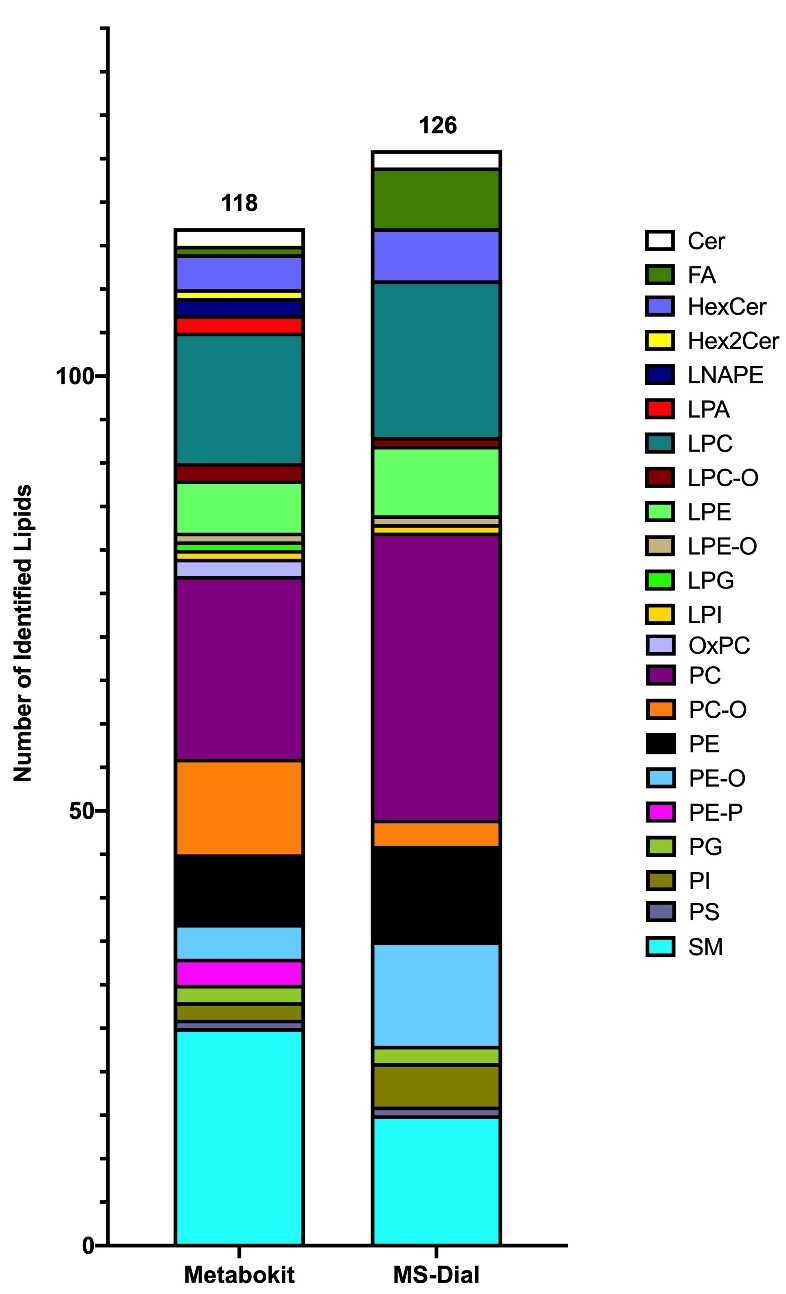
**

**Figure S2:** Identification of lipid species using MetaboKit vs MS-Dial for (a) Positive Mode and (b) Negative Mode when analysing samples with iterative MSMS (10 iterative injections) and DDA, using the same search space (spectral libraries). Lipid coverage of MetaboKit was comparable to MS-Dial for both polarities (219 vs 216 identified lipids in positive mode, 118 vs 126 identified lipids in negative mode).

**a**


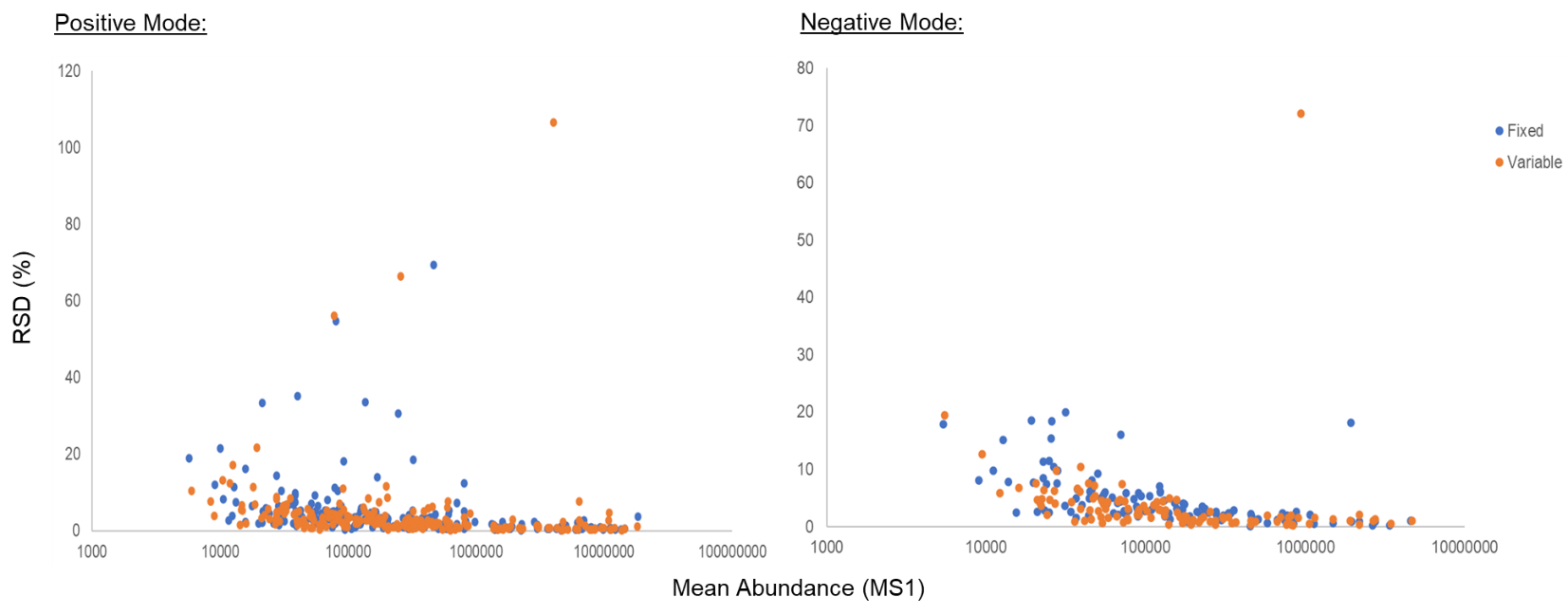


**b**


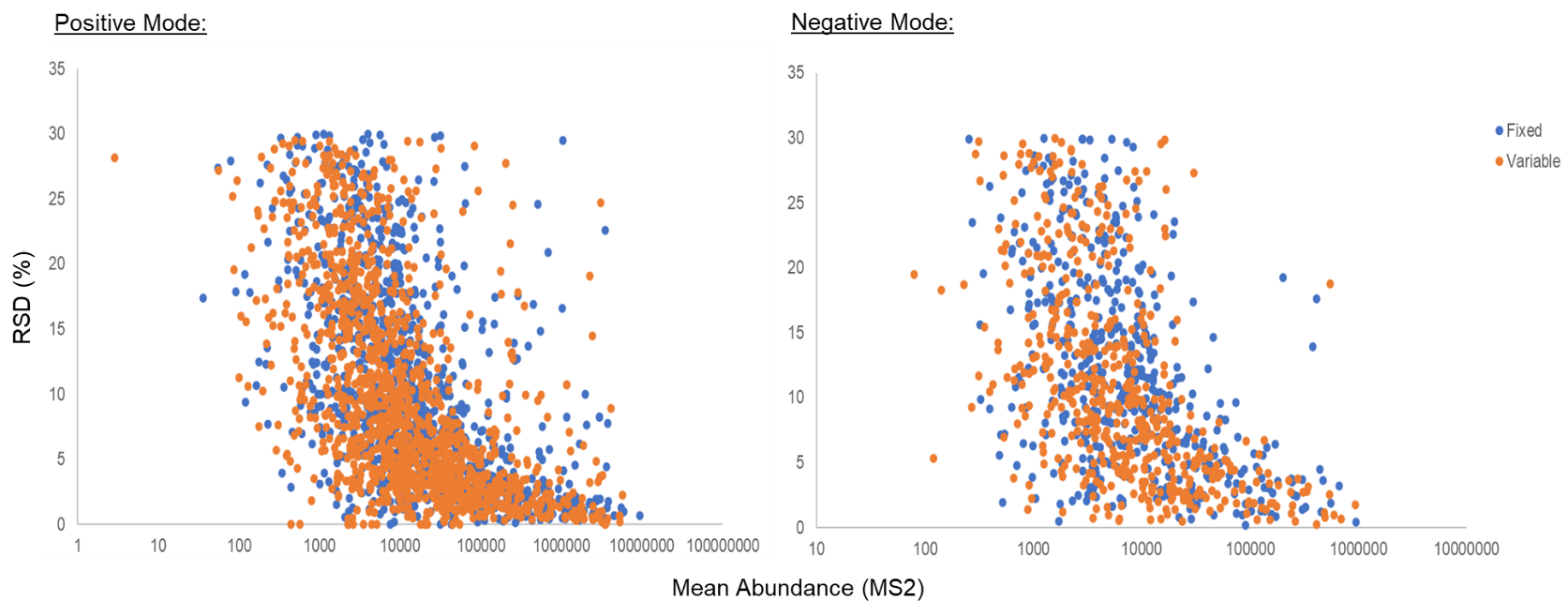


**Figure S3:** RSD (%) vs mean abundance in DIA MS1 (a) and RSD (%) vs mean abundance in DIA MS2 (b) in positive and negative mode. As expected, the value of RSD (%) generally decreases with increasing signal intensity.


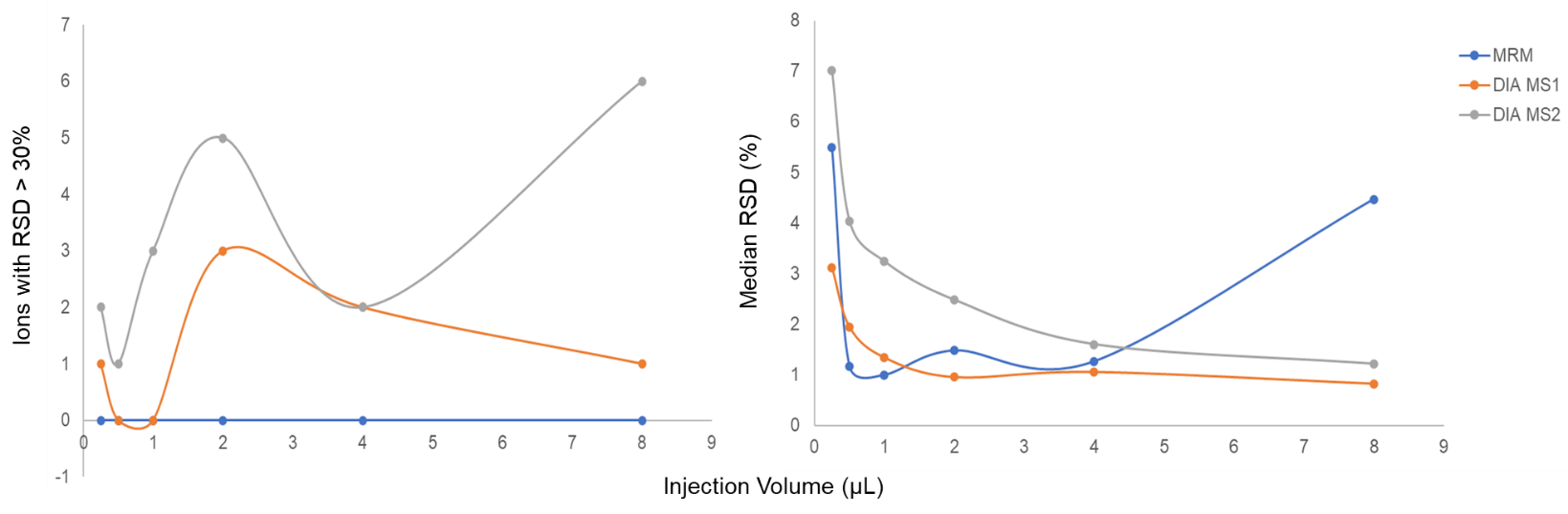


**Figure S4**: RSD (%) vs injection volume for same lipid species in MRM and DIA modes. In our experimental conditions, an injection volume of around 4 µL of lipid extract was the best choice for DIA experiments, as it returned the smallest number of ions with RSD > 30% and lowest median RSD when taking into account both MS1 and MS2 levels.


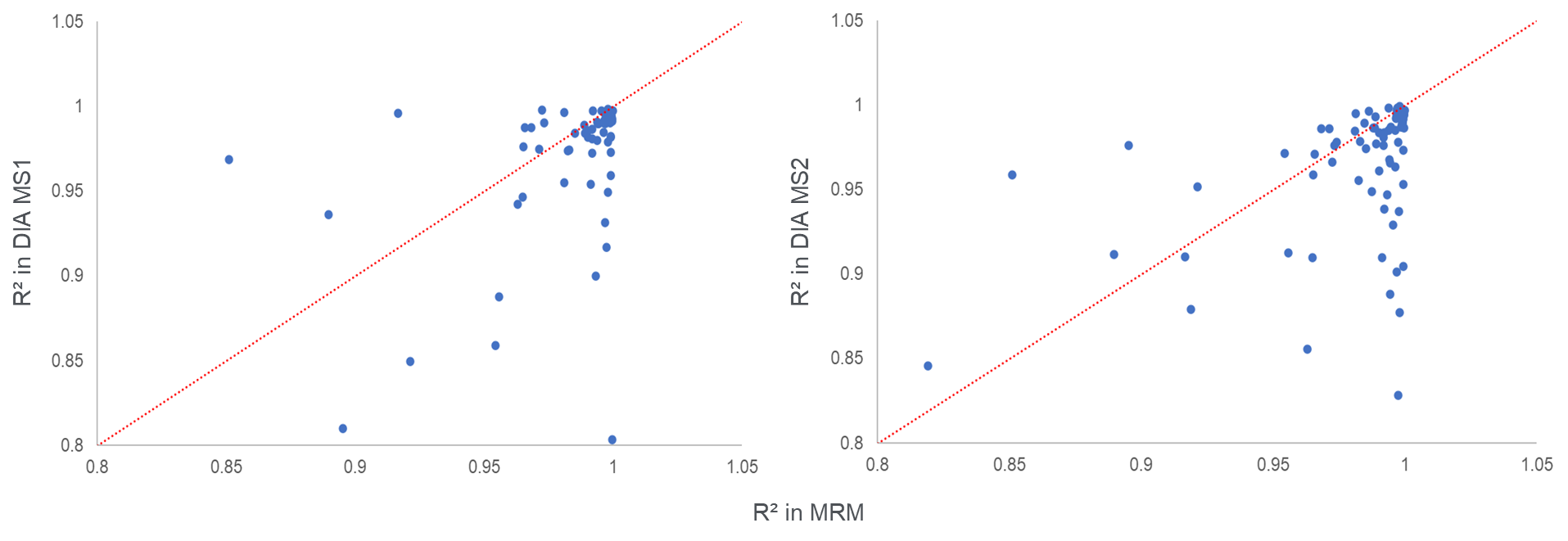


**Figure S5**: Comparison between the R^2^ values from DIA MS2 fragments and DIA MS1 features with the R^2^ values of the corresponding MRM transitions (only for R^2^ > 0.8) to evaluate the quantification performances of the different approaches.


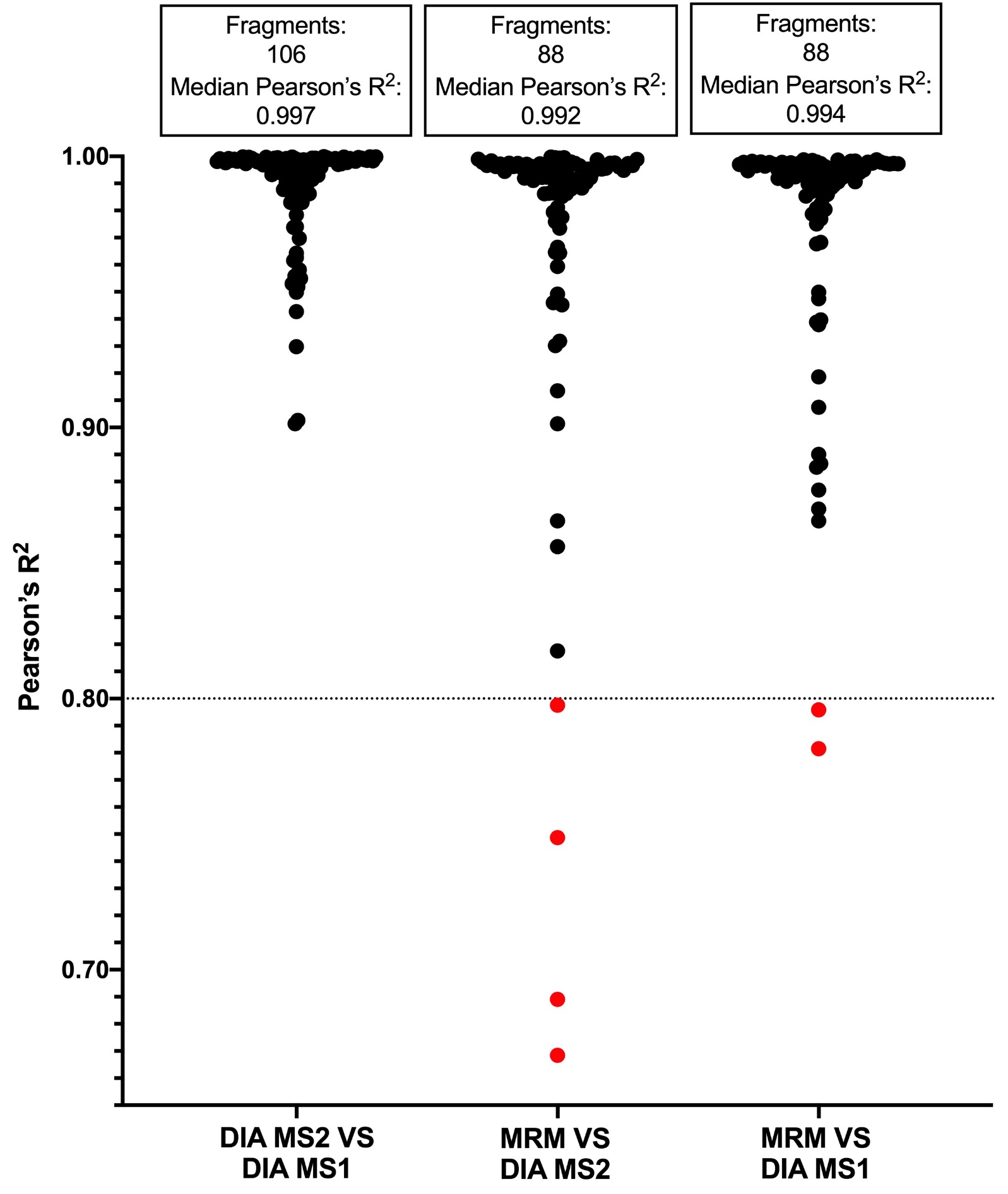


**Figure S6**: Dot plot of Pearson’s R^2^ representing signal intensities of DIA MS2 and MRM transitions in pooled extracts from commercial plasma. DIA MS2 fragments are highly correlated with DIA MS1 features and MRM transitions. Comparison of DIA MS2 vs DIA MS1 was done for all the 106 quantified lipids, using the DIA MS2 fragment with the highest Pearson’s correlation for each lipid, while comparison of MRM vs DIA was done only for the 88 lipids that were detected in both acquisition approaches. Red data points indicate comparisons with Pearson’s R^2^ < 0.8.


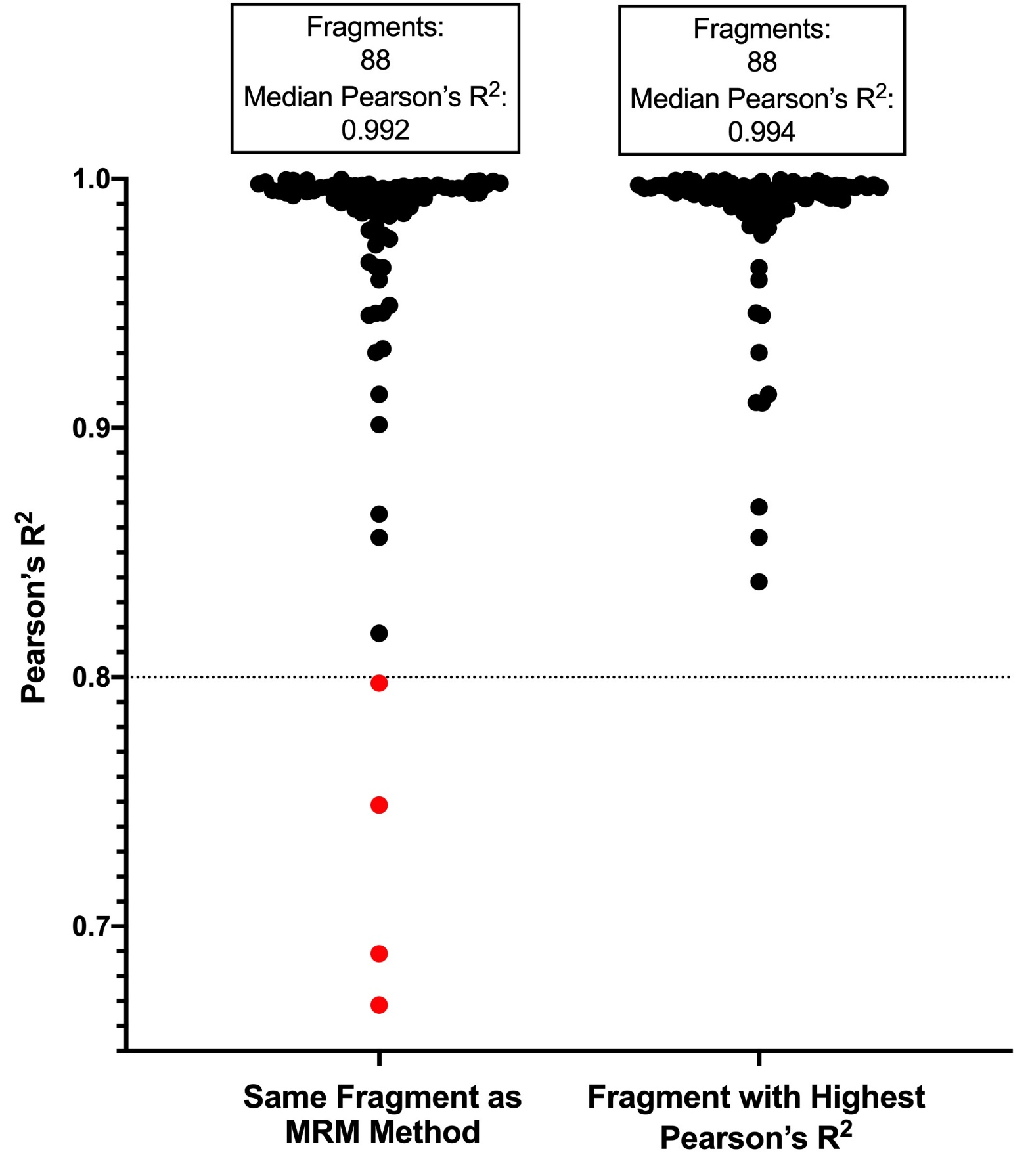


**Figure S7**: Pearson’s correlation between the levels of DIA MS2 fragments that were also measured by MRM, or the DIA MS2 fragments with the highest Pearson’s R^2^, with their corresponding MRM features for 106 plasma lipids. Overall, Pearson’s R^2^ was higher when the DIA MS2 fragments with highest correlation against their corresponding MRM features were used. Red data points indicate comparisons with Pearson’s R^2^ < 0.8.

**a b c**


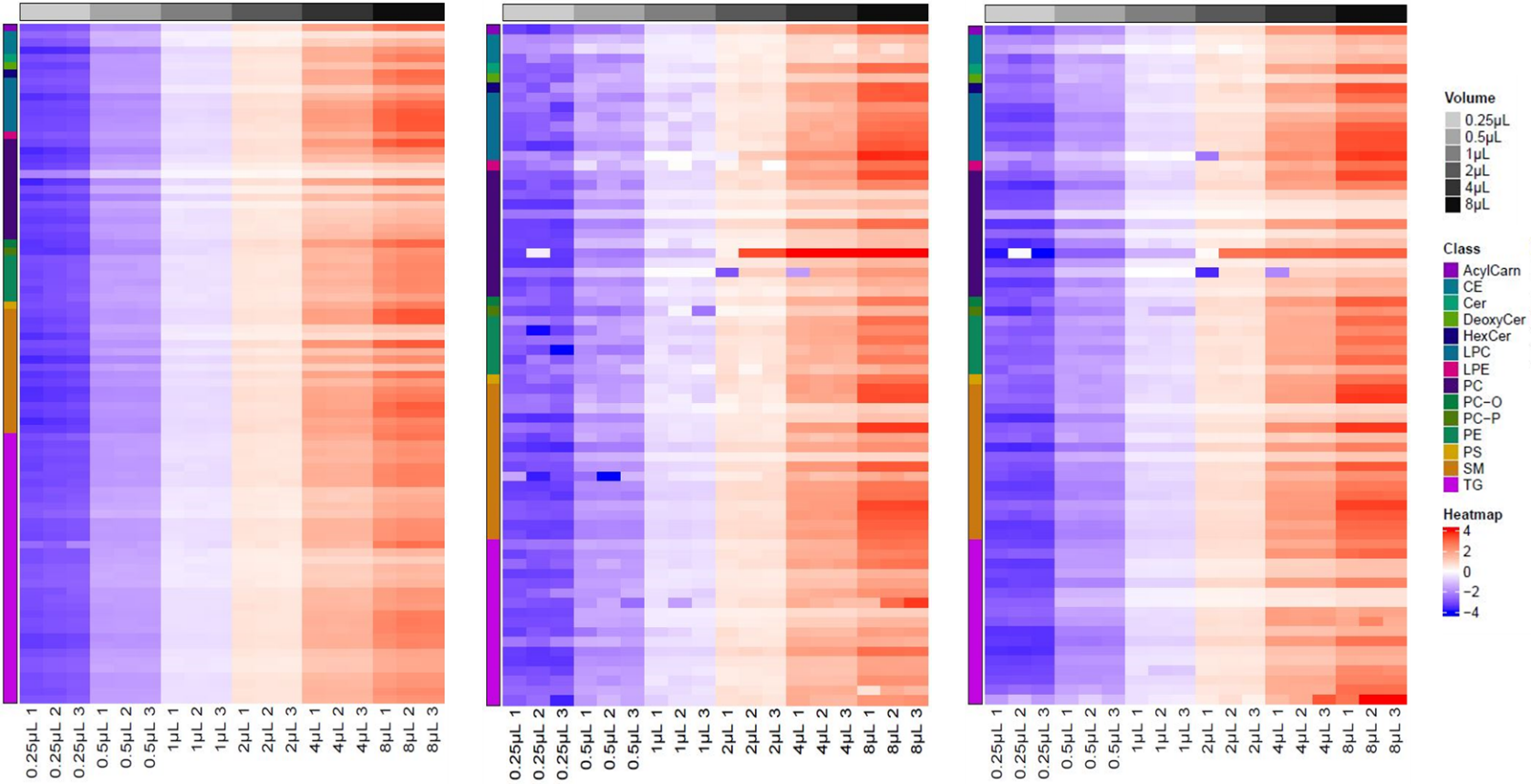


**Figure S8**: Heatmaps of normalized median-centred fold changes when using MRM (a), DIA MS2 (b) and DIA MS1 (c) modes for the lipids identified and quantified in dilution series of extracts from commercial human plasma. A total of 88 fragments were measured and compared in MRM, DIA MS2 and DIA MS1. A proportional increase in the level of all lipids was seen with an increase in injection volume (grey to black) for MRM, DIA MS2 and DIA MS1, showing comparable quantitation performances between the methods.

**a**


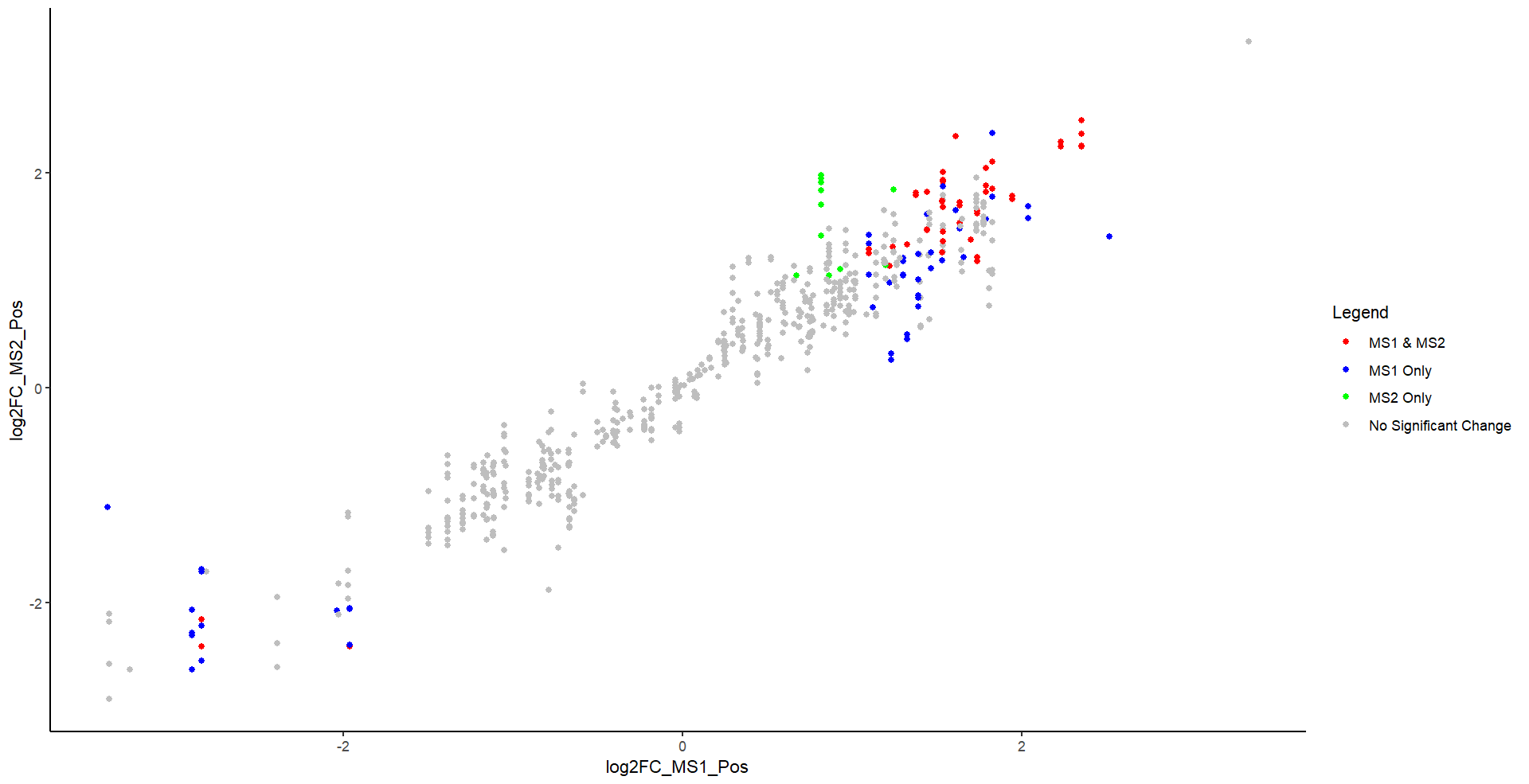


**b**


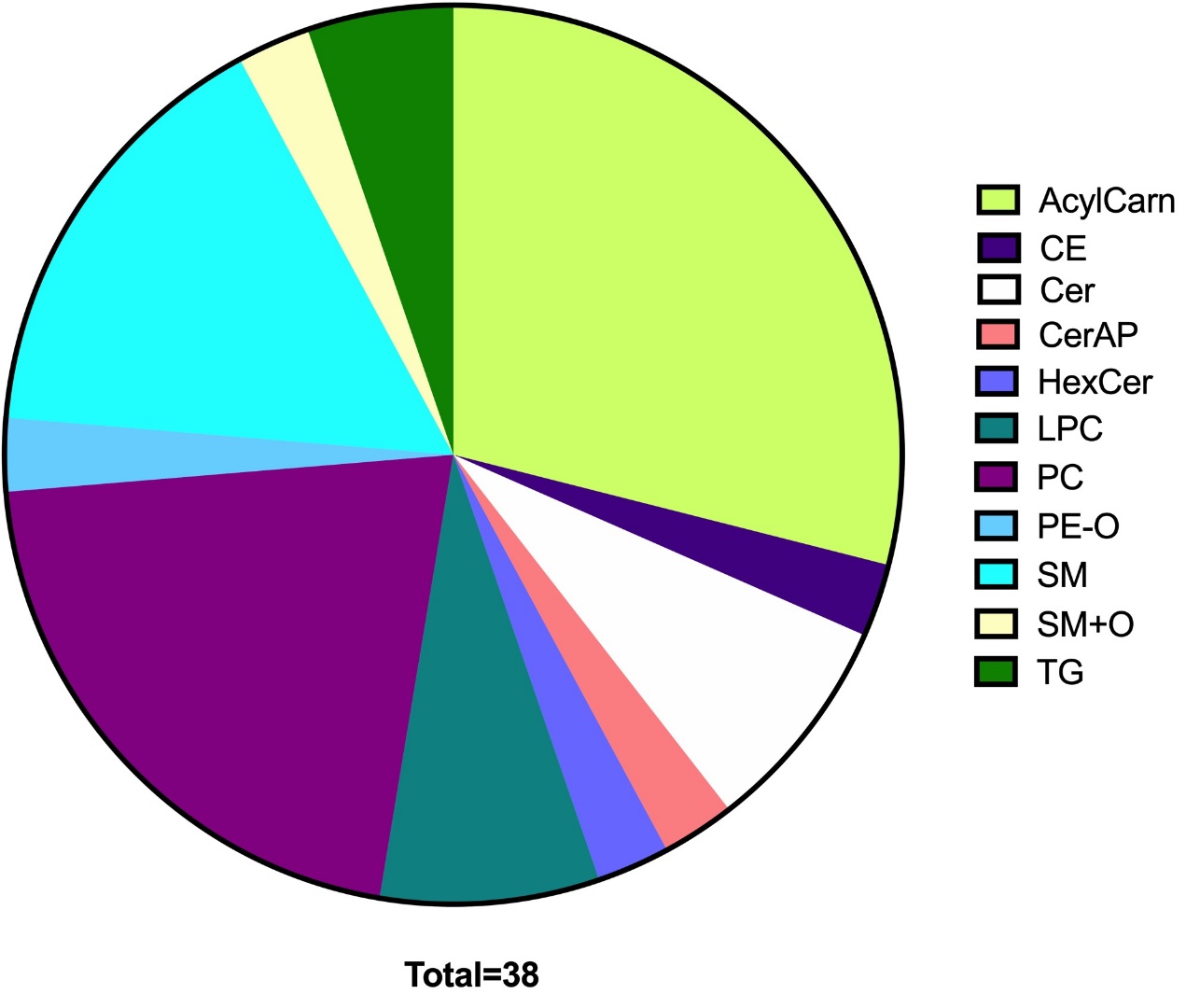


**c**


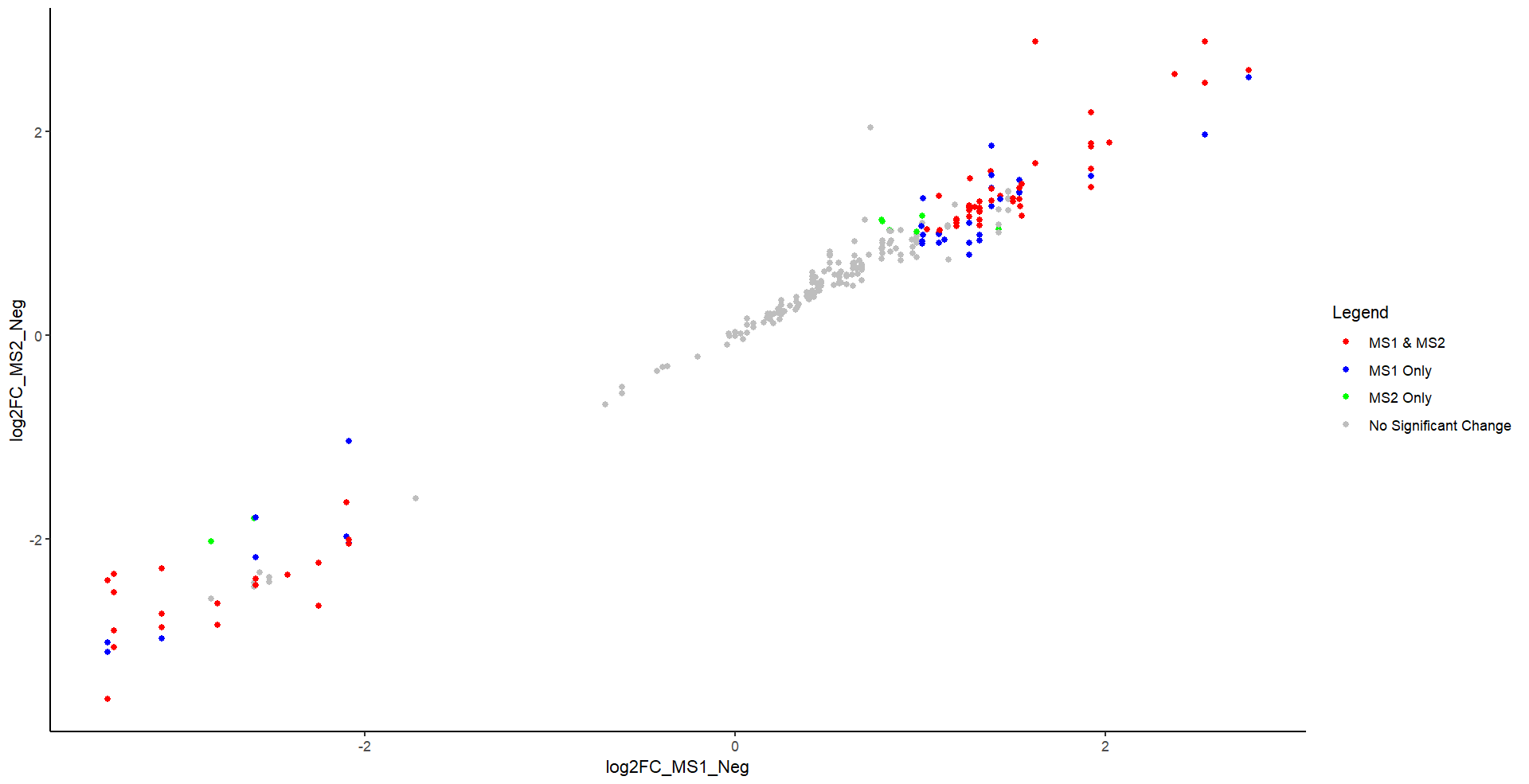


**d**


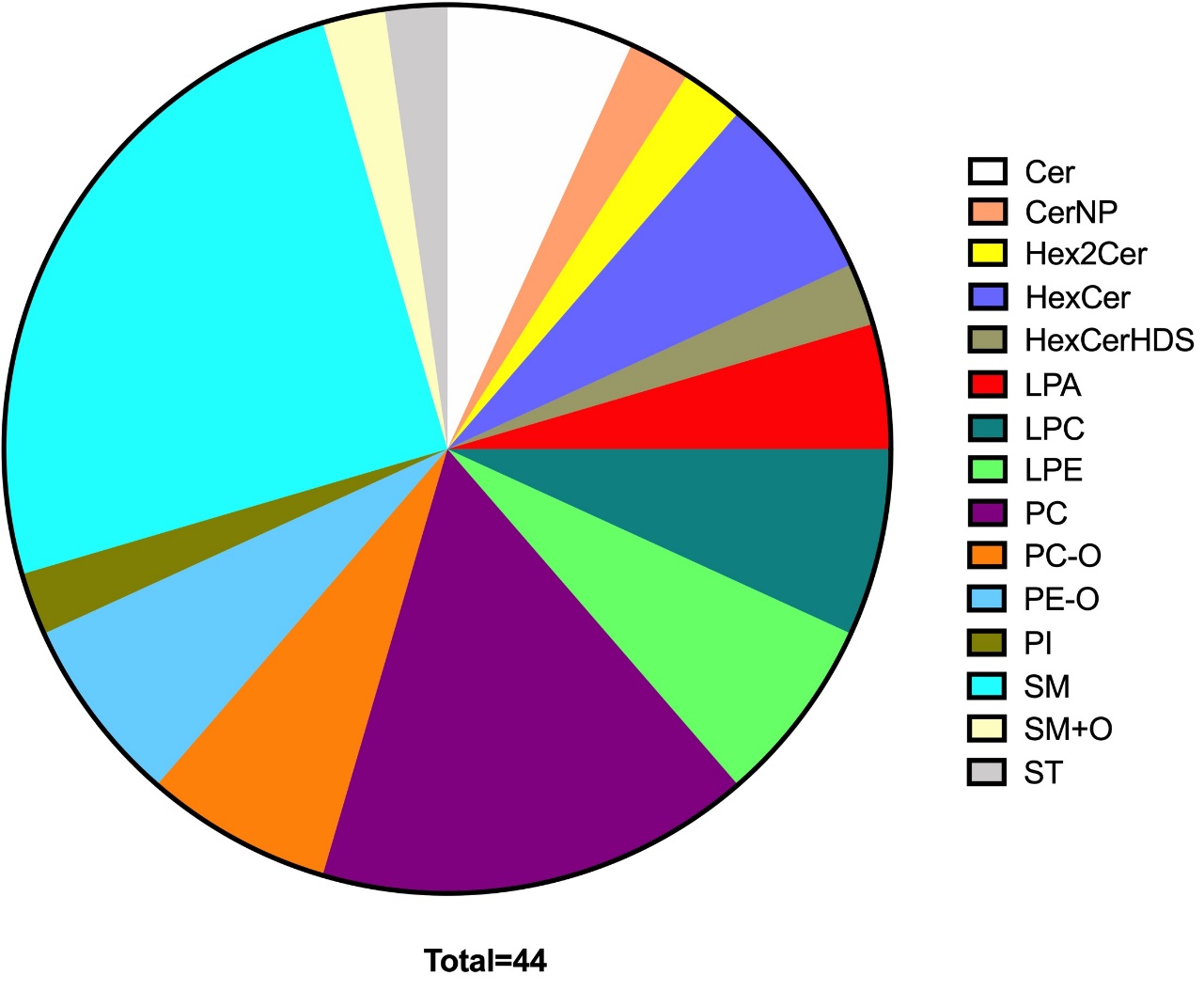


**Figure S9**: Differential expression analysis results of WT vs CerS2 null mice for 173 serum lipid species in Positive Mode (a) and 119 lipids in Negative Mode (c). In both polarities, most lipid species present at significantly different levels between WT and CerS2 null mice showed the same behaviour when considering MS1 or MS2 signals (FC > 2, FDR < 0.05). Of these, 38 and 44 molecular species were found to be significantly different between WT and CerS2 null mice in positive mode and negative mode respectively, with 19 different lipid classes represented across both polarities (b,d).

**a b**


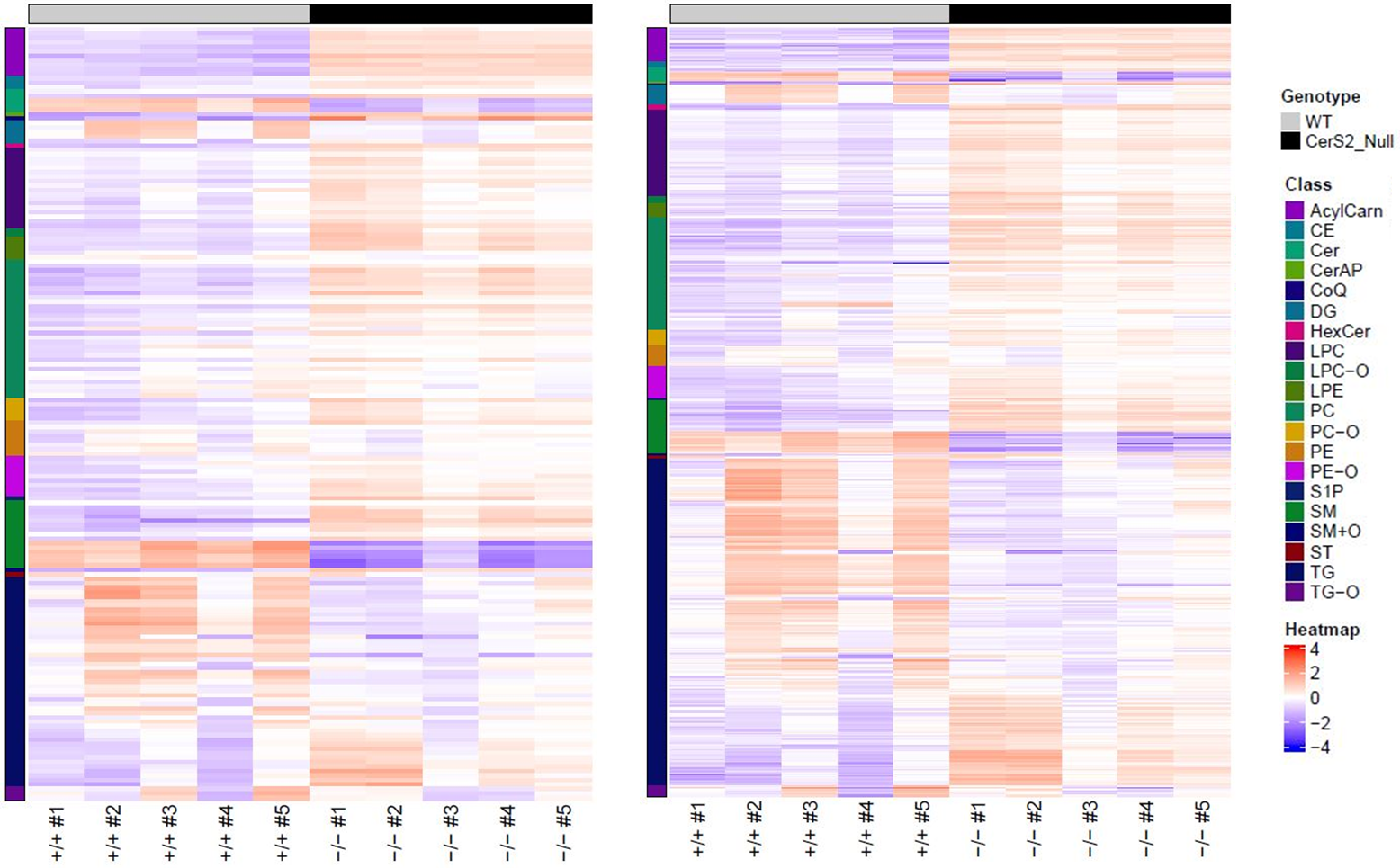


**c d**

**
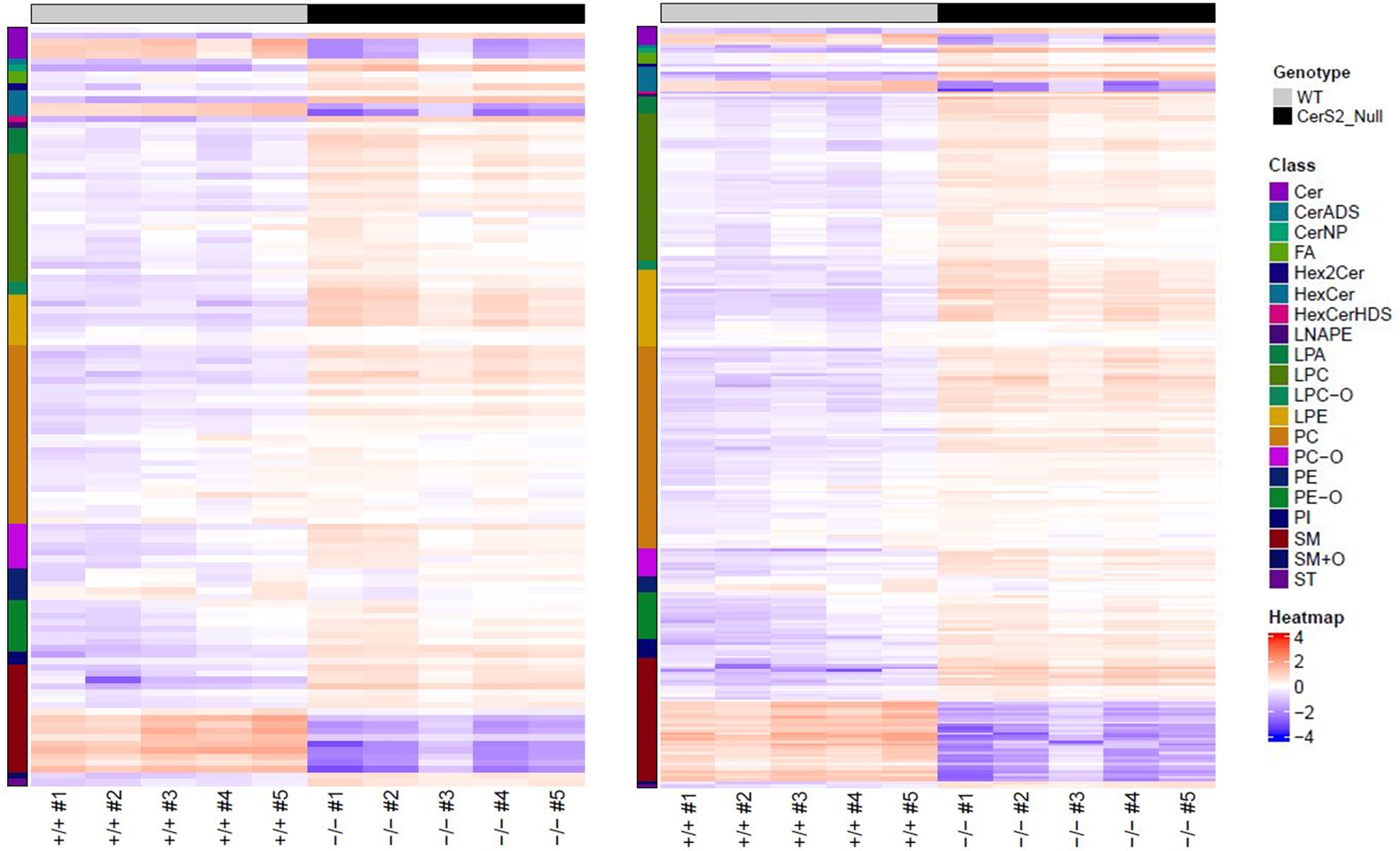
**

**Figure S10**: Heatmaps of normalized median-centred fold changes of lipids in WT vs CerS2 null mice serum samples in DIA MS1 Positive Mode (a), DIA MS2 Positive Mode (b), DIA MS1 Negative Mode (c) and DIA MS2 Negative Mode (d). WT (grey) and CerS2 null samples (black) clearly showed a different serum lipidomic profile, confirmed using quantitation at both MS1 and MS2 levels.

Table S1: Values of fixed Q1 windows used in DIA experiments.

| **Experiment** | **Positive/Negative Mode** | | |
| --- | --- | --- | --- |
|  | m/z start | m/z end | Window Length |
| TOF-MS | 300 | 1,000 | N/A |
| SWATH-MS/MS 1 | 300 | 320 | 20 |
| SWATH-MS/MS 2 | 319 | 340 | 21 |
| SWATH-MS/MS 3 | 339 | 360 | 21 |
| SWATH-MS/MS 4 | 359 | 380 | 21 |
| SWATH-MS/MS 5 | 379 | 400 | 21 |
| SWATH-MS/MS 6 | 399 | 420 | 21 |
| SWATH-MS/MS 7 | 419 | 440 | 21 |
| SWATH-MS/MS 8 | 439 | 460 | 21 |
| SWATH-MS/MS 9 | 459 | 480 | 21 |
| SWATH-MS/MS 10 | 479 | 500 | 21 |
| SWATH-MS/MS 11 | 499 | 520 | 21 |
| SWATH-MS/MS 12 | 519 | 540 | 21 |
| SWATH-MS/MS 13 | 539 | 560 | 21 |
| SWATH-MS/MS 14 | 559 | 580 | 21 |
| SWATH-MS/MS 15 | 579 | 600 | 21 |
| SWATH-MS/MS 16 | 599 | 620 | 21 |
| SWATH-MS/MS 17 | 619 | 640 | 21 |
| SWATH-MS/MS 18 | 639 | 660 | 21 |
| SWATH-MS/MS 19 | 659 | 680 | 21 |
| SWATH-MS/MS 20 | 679 | 700 | 21 |
| SWATH-MS/MS 21 | 699 | 720 | 21 |
| SWATH-MS/MS 22 | 719 | 740 | 21 |
| SWATH-MS/MS 23 | 739 | 760 | 21 |
| SWATH-MS/MS 24 | 759 | 780 | 21 |
| SWATH-MS/MS 25 | 779 | 800 | 21 |
| SWATH-MS/MS 26 | 799 | 820 | 21 |
| SWATH-MS/MS 27 | 819 | 840 | 21 |
| SWATH-MS/MS 28 | 839 | 860 | 21 |
| SWATH-MS/MS 29 | 859 | 880 | 21 |
| SWATH-MS/MS 30 | 879 | 900 | 21 |
| SWATH-MS/MS 31 | 899 | 920 | 21 |
| SWATH-MS/MS 32 | 919 | 940 | 21 |
| SWATH-MS/MS 33 | 939 | 960 | 21 |
| SWATH-MS/MS 34 | 959 | 980 | 21 |
| SWATH-MS/MS 35 | 979 | 1000 | 21 |

Table S2: Values of the size of Q1 variable windows used for DIA in positive and negative ionization modes*

| **Experiment** | **Positive mode** | | | **Negative mode** | | |
| --- | --- | --- | --- | --- | --- | --- |
|  | m/z start | m/z end | Window Length | m/z start | m/z end | Window Length |
| TOF-MS | 300.00 | 1000.00 | N/A | 300.00 | 1000.00 | N/A |
| SWATH-MS/MS 1 | 300.00 | 398.83 | 98.83 | 300.00 | 391.79 | 91.79 |
| SWATH-MS/MS 2 | 397.83 | 469.89 | 72.06 | 390.79 | 475.88 | 85.09 |
| SWATH-MS/MS 3 | 468.89 | 540.95 | 72.06 | 474.88 | 558.96 | 84.08 |
| SWATH-MS/MS 4 | 539.95 | 595.01 | 55.07 | 557.96 | 634.83 | 76.87 |
| SWATH-MS/MS 5 | 594.01 | 649.08 | 55.07 | 633.83 | 681.08 | 47.24 |
| SWATH-MS/MS 6 | 648.08 | 723.01 | 74.93 | 680.08 | 710.00 | 29.93 |
| SWATH-MS/MS 7 | 722.01 | 752.06 | 30.05 | 709.00 | 734.08 | 25.08 |
| SWATH-MS/MS 8 | 751.06 | 775.06 | 24.00 | 733.08 | 760.26 | 27.18 |
| SWATH-MS/MS 9 | 774.06 | 793.05 | 18.99 | 759.26 | 778.06 | 18.80 |
| SWATH-MS/MS 10 | 792.05 | 807.15 | 15.09 | 777.06 | 800.17 | 23.11 |
| SWATH-MS/MS 11 | 806.15 | 826.18 | 20.04 | 799.17 | 819.06 | 19.89 |
| SWATH-MS/MS 12 | 825.18 | 844.23 | 19.04 | 818.06 | 834.94 | 16.89 |
| SWATH-MS/MS 13 | 843.23 | 866.13 | 22.90 | 833.94 | 847.08 | 13.13 |
| SWATH-MS/MS 14 | 865.13 | 883.26 | 18.13 | 846.08 | 863.10 | 17.02 |
| SWATH-MS/MS 15 | 882.26 | 898.26 | 16.00 | 862.10 | 875.17 | 13.07 |
| SWATH-MS/MS 16 | 897.26 | 913.37 | 16.11 | 874.17 | 893.03 | 18.87 |
| SWATH-MS/MS 17 | 912.37 | 927.32 | 14.94 | 892.03 | 912.16 | 20.12 |
| SWATH-MS/MS 18 | 926.32 | 946.29 | 19.97 | 911.16 | 934.13 | 22.98 |
| SWATH-MS/MS 19 | 945.29 | 965.21 | 19.93 | 933.13 | 958.24 | 25.11 |
| SWATH-MS/MS 20 | 964.21 | 1000.00 | 35.79 | 957.24 | 1000.00 | 42.76 |

* Variable windows were determined using DDA data of a QC sample ran on a variable window calculator in-built in MetaboKit.

Table S3: Descriptive statistics of Pearson’s R^2^ values for the correlation between DIA MS2 and DIA MS1 measurements when considering all the DIA MS2 fragments or only the ones with the highest R^2^ with their corresponding DIA MS1 features.

|  | **DIA MS2 vs DIA MS1** | |
| --- | --- | --- |
|  | **All DIA MS2 Fragments** | **DIA MS2 Fragments with Highest Pearson's Correlation with DIA MS1** |
| Total Number of Fragments | 651 | 106 |
| Median Pearson's R² | 0.973 | 0.997 |
| Number of Fragments with Pearson's R² < 0.8 | 126 (19.35%) | 0 (0%) |
|  |  |  |

Table S4: Variable Q1 windows used in DIA mode to measure plasma lipids in WT and CerS2 Null mouse serum in positive and negative ionization modes*

| **Experiment** | **Positive mode** | | | **Negative mode** | | |
| --- | --- | --- | --- | --- | --- | --- |
|  | m/z start | m/z end | Window Length | m/z start | m/z end | Window Length |
| TOF-MS | 300.00 | 1000.00 | N/A | 300.00 | 1000.00 | N/A |
| SWATH-MS/MS 1 | 298.50 | 365.66 | 67.16 | 298.50 | 377.77 | 79.27 |
| SWATH-MS/MS 2 | 364.66 | 431.82 | 67.16 | 376.77 | 431.68 | 54.92 |
| SWATH-MS/MS 3 | 430.82 | 504.81 | 73.99 | 430.68 | 495.84 | 65.15 |
| SWATH-MS/MS 4 | 503.81 | 546.85 | 43.05 | 494.84 | 543.94 | 49.10 |
| SWATH-MS/MS 5 | 545.85 | 583.82 | 37.97 | 542.94 | 594.08 | 51.15 |
| SWATH-MS/MS 6 | 582.82 | 639.07 | 56.25 | 593.08 | 656.06 | 62.98 |
| SWATH-MS/MS 7 | 638.07 | 695.86 | 57.79 | 655.06 | 700.13 | 45.07 |
| SWATH-MS/MS 8 | 694.86 | 743.07 | 48.22 | 699.13 | 728.05 | 28.91 |
| SWATH-MS/MS 9 | 742.07 | 767.16 | 25.08 | 727.05 | 750.03 | 22.99 |
| SWATH-MS/MS 10 | 766.16 | 787.14 | 20.98 | 749.03 | 768.04 | 19.01 |
| SWATH-MS/MS 11 | 786.14 | 802.22 | 16.08 | 767.04 | 784.11 | 17.07 |
| SWATH-MS/MS 12 | 801.22 | 818.15 | 16.93 | 783.11 | 802.05 | 18.94 |
| SWATH-MS/MS 13 | 817.15 | 833.09 | 15.94 | 801.05 | 818.00 | 16.95 |
| SWATH-MS/MS 14 | 832.09 | 847.17 | 15.08 | 817.00 | 830.08 | 13.08 |
| SWATH-MS/MS 15 | 846.17 | 864.15 | 17.99 | 829.08 | 844.16 | 15.08 |
| SWATH-MS/MS 16 | 863.15 | 888.29 | 25.14 | 843.16 | 857.10 | 13.95 |
| SWATH-MS/MS 17 | 887.29 | 913.16 | 25.87 | 856.10 | 870.15 | 14.04 |
| SWATH-MS/MS 18 | 912.16 | 939.35 | 27.20 | 869.15 | 891.18 | 22.04 |
| SWATH-MS/MS 19 | 938.35 | 960.38 | 22.03 | 890.18 | 917.20 | 27.02 |
| SWATH-MS/MS 20 | 959.38 | 999.50 | 40.12 | 916.20 | 998.50 | 82.30 |

* Variable windows were determined using DDA data of a QC sample ran on a variable window calculator in-built in MetaboKit

Table S5: Descriptive statistics of DIA data for all lipid species quantified in all technical replicates of QC, WT and CerS2 null mouse serum samples*

|  | **Positive Mode** | | |  | **Negative Mode** | | |
| --- | --- | --- | --- | --- | --- | --- | --- |
|  | **QC (n=6)** | **WT (n=5)** | **CerS2 Null (n=5)** |  | **QC (n=6)** | **WT (n=5)** | **CerS2 Null (n=5)** |
| **MS1** |  |  | | | | | |
|  |  |  |  |  |  |  |  |
| Total Number of Features |  | 244 |  |  | 143 | | |
| Median RSD (%) | 7.41 | 22.74 | 15.99 |  | 5.23 | 16.02 | 11.53 |
| Number of MS1 Features with RSD 20-30% | 7 | 80 | 46 |  | 2 | 5 | 10 |
| Number of MS1 Features with RSD < 20% | 233 | 97 | 167 |  | 140 | 35 | 122 |
| **MS2** |  |  |  |  |  |  |  |
|  |  |  |  |  |  |  |  |
| Total Number of Quantified MS2 Fragments |  | 1844 |  |  |  | 755 |  |
| Median RSD (%) | 17.72 | 34.00 | 25.63 |  | 18.13 | 25.02 | 21.50 |
| Number of MS2 Transitions with RSD 20-30% | 281 | 435 | 432 |  | 152 | 192 | 151 |
| Number of MS2 Transitions with RSD < 20% | 1011 | 315 | 642 |  | 408 | 267 | 347 |
| Number of MS2 Fragments with Pearson's R² < 0.8 | 1168 (63.34% of all quantified transitions) | | |  | 459 (60.79% of all quantified MS2 fragments) | | |
| Number of Lipids with MS1 & MS2 that Passed Filtering | 173 | | |  | 119 | | |

*Each of the 5 biological replicates of WT and CerS2 null mouse serum was injected in triplicates during data acquisition and all injections were included for computation of RSD.
